# Self-reported and parent-reported mental health in children from low-income families in Agder, Norway: results from baseline measurements of New Patterns project participants

**DOI:** 10.1136/bmjopen-2023-076400

**Published:** 2023-11-27

**Authors:** Tormod Bøe, Helene Angelica Ostojic, Kristin Haraldstad, Eirik Abildsnes, Philip Wilson, Kristine Vigsnes, Eirin Mølland

**Affiliations:** 1 Department of Psychosocial Science, University of Bergen, Bergen, Norway; 2 Regional Centre for Child and Youth Mental Health and Child Welfare, NORCE Norwegian Research Centre AS Forskningsområde Helse, Bergen, Norway; 3 Drammen District Psychiatric Center, Mental Health and Substance Abuse, Vestre Viken HF, Drammen, Norway; 4 Department of Health and Nursing Sciences, Faculty of Health and Sport Sciences, University of Agder, Kristiansand, Norway; 5 Institute of Health and Society, University of Oslo, Oslo, Norway; 6 Centre for Rural Health, University of Aberdeen, Aberdeen, UK; 7 Kristiansand Kommune, Kristiansand, Norway; 8 School of Business and Law, University of Agder, Kristiansand, Norway; 9 NORCE Norwegian Research Centre AS Forskningsomrade Samfunn, Bergen, Norway

**Keywords:** SOCIAL MEDICINE, Health Equity, MENTAL HEALTH

## Abstract

**Background:**

Poverty may pose risks to child and adolescent mental health, but few studies have reported on this association among children and adolescents in low-income families in Norway.

**Methods:**

Based on a sample participating in an intervention for low-income families in Norway, we report data from the survey administered at the start of the intervention. Mental health problems were measured using the Strengths and Difficulties Questionnaire (SDQ; self-report (SR) n = 148; parent/proxy-report (PR) n = 153, mean age = 10.8). Demographic and family characteristics were obtained from parent reported data. Results are presented by gender and migration background. Regression analysis was used to investigate the relative contribution of background factors to mental health symptoms. The distribution of scores is compared to UK norms.

**Results:**

Participants reported relatively high scores on the Strengths and Difficulties Questionnaire (SDQ) Total Difficulties Scale (parent/proxy-report, PR mean=10.7; self-report, SR mean=10.1). Participants with non-immigrant backgrounds scored considerably higher on the Total Difficulties Scale (PR mean difference=2.9; SR 5.3) and on most other domains measured with the SDQ compared with their peers with immigration backgrounds. Participants generally scored higher than or equal to UK norms.

**Conclusion:**

Participants in the current study had many symptoms of mental health problems, with large differences between those with and without a migrant background. Interventions for low-income families should be based on detailed knowledge about differences in family risks, resources and needs.

**STRENGTHS AND LIMITATIONS OF THIS STUDY**

- This study presents baseline data on mental health problems gathered using a validated instrument among participants in a poverty intervention study conducted in southern Norway.
- The study provides valuable information about a hard-to-reach sample and includes participants with and without immigrant backgrounds.
- Due to recruitment procedures, there is no control or comparison group included in the survey.
- The recruitment procedure may have biased the sample towards poor families with many difficulties, making the findings somewhat less generalisable.

## Introduction

Mental health problems in children and adolescents are common. Up to 20% of children and young adolescents have a mental disorder,^1^ and the prevalence may be rising, for girls in particular.^2^ Mental health problems negatively impact young people’s health and well-being, as well as their future educational prospects, employment and earnings.^3^ Mental health problems also account for a substantial and increasing worldwide burden of ill health.^4^

Child and adolescent mental health problems are distributed unequally with regard to household income: more children and adolescents in families with low income have mental health problems compared with more affluent peers.^5–7^ Children and adolescents with low socioeconomic status (SES) are two to three times more likely to develop mental health problems than their peers with high SES.^5 8^ Research suggests low income mainly influences children and adolescents indirectly, through constraining caregivers’ abilities to deploy resources towards their development, and through increased family stress with detrimental consequences for parental mental health and parenting practices.^9^ There may also be direct influences through toxic effects of poverty-related stress on the brains of children and adolescents, potentially influencing structure and function in brain areas involved in memory, emotion regulation and executive function, with implications for mental health and cognitive functioning.^10^

Migration background may also be related to elevated mental health problems in children and adolescents.^11^ It was, for example, found that second-generation migrant children had higher rates of depression, anxiety and post-traumatic stress disorders compared with their native counterparts.^12^ This is particularly relevant to the current study, as migrant families and their children make up a large portion of the low-income population in Norway.^13^

The current study is based on data from New Patterns, an intervention study conducted in a sample of families with children younger than 17 purposefully recruited based on having household incomes below the low-income threshold in Norway (ie, ≤60% of the equivalised population median income) as well as having long-standing needs for welfare services.^14^ Families that are included in New Patterns receive a family coordinator who is responsible for follow-up of all family members for a period of 5 years and coordinate services that are involved with the family. Norwegian register-based and community-based observational studies have shown that children and adolescents from households with lower income and low SES have a higher frequency of mental disorders relative to higher income peers.^15^ Many children and adolescents with mental health problems nevertheless go undetected.^16^ Furthermore, children from low-income families are less likely to participate in research and community programmes compared with more affluent peers.^17^ Therefore, register-based studies should be complemented by epidemiological studies directly targeting these low-income hard-to-reach samples to more fully capture the mental health status of children and adolescents who live in low-income families.

The aim of the study is to present results from the baseline mental health screening completed by participants in the New Patterns project. In presenting these data, we will provide results separately by informant; for younger children, their parent or a proxy completed the Strengths and Difficulties Questionnaire (SDQ), but older children completed it themselves. We also stratify our results by sex as differences in the trajectories of mental health problems among boys and girls are widely reported.^18^ Finally, we present results separately for children and adolescents with and without immigrant background since previous studies have shown that ethnic minority children have higher mean SDQ scores compared with majority population samples in Norway.^19^

## Methods

### Participants and procedures

The New Patterns project targets families with children aged 0–17 living in Norway with low household income and additional challenges requiring long-standing need for services.^14^ Families were referred to the intervention from different service sectors within the municipalities (ie, kindergarten, school, public health clinics, general practitioners, the Norwegian Labour and Welfare Administration, child protection services and mental health services). Each referral was subsequently discussed in a multidisciplinary intake team who selected the families that were considered eligible to participate based on their needs for coordinated longer-term follow-up.

Our sample consists of the baseline self-reported (number of participants (n)=148, age mean (M)=13.55, SD=1.95) and parent-reported (n=153, age M=8.07, SD=3.01) SDQ questionnaires for children participating in the New Patterns project. The sample was restricted to reports that were gathered within 360 days after enrolment to avoid potentially measuring the effect of the intervention and for children in the age range where SDQ is validated (4–17 years in the current study). The median time in the project when the SDQ was reported was 82 days. Twelve children were included in both the parent-reported and self-reported data. As results are presented by informants, this has not resulted in any double counting or other violations of sound analytical nor statistical practices.

### Patient and public involvement

Patients and/or the public were not involved in the design, or conduct, or reporting, or dissemination plans of this research.

### Instruments

#### Mental health problems

Mental health was assessed with parent-report and self-report versions of the extended Norwegian translation of the SDQ.^20^ The psychometric properties of the SDQ have been well documented.^21 22^ The Norwegian translations of the SDQ have shown strong psychometric properties for all informant versions (abstract in English available at the PSYKTESTBARN website: shorturl.at/DUVY2), and have also received support for administration to 18–19 years.^23^ The SDQ contains 25 items, each of which can be answered using: ‘certainly true’, ‘somewhat true’ or ‘not true’. They can be summed into five subscales each containing five items with a potential range of scores from 0 to 10: conduct problems, hyperactivity/inattention, emotional problems, peer relationship problems and prosocial behaviour. The first four subscales can be added together as a Total Difficulties Scale, with a potential range from 0 to 40. The extended SDQ includes an impact supplement with items measuring functional impairment regarding chronicity, distress, social impairment and burden to others.^20^

#### Sociodemographic variables

Background characteristics were measured when the family entered the New Patterns project. Parental education was assessed as the highest level of education completed by the mother or father. If at least one of the parents in the household was employed at the time of entering the project, we defined parental employment status to be employed. Children who were born in Norway with one or two parents who had immigrated to Norway or children who themselves had immigrated to Norway were defined as having an immigrant background. Children born in Norway whose parents did not have immigrant background were defined as having a non-immigrant background. We used information about year of birth and the date of answering the SDQ questionnaire to compute approximately the age at which the SDQ questionnaire was completed.

#### Statistical analyses

Mean differences with 95% CIs in SDQ scores between boys and girls, and between children with immigrant and non-immigrant background were analysed using t-tests assuming unequal variance. Self-reported and parent-reported data were analysed separately. We then proceeded to estimate the following regression model:

$$y_{i}=\beta_{0}+\beta_{1}girl_{i}+\beta_{2}age_{i}+\beta_{3}Immigrant_{i}+\beta_{4}Time_{i}+\epsilon_{i}$$

Where $y_{i}$ is the outcome variable; SDQ Total Difficulties Scale score and the five subscales. The variable girl is a dummy variable indicating if the individual is a girl, age is a continuous variable measuring age in years at the time when SDQ was reported. This variable was mean centred in the analyses (age_centred_=age–mean(age)) to ease interpretability of the regression coefficients. Immigrant is a dummy variable indicating if the child has immigrant background. Time is a continuous variable measuring the number of days since a family coordinator was assigned to the family. Robust standard errors were clustered on the family level. Stata V.17 was used for all analyses^24^ whereas the R-package ggplot2^25^ was used for making figures.

To compare the distribution of scores in our current sample with UK norms, we created cut-points corresponding to the UK 4-band solution.^26^ These cut-points are based on percentile distributions in a large UK sample and use the categories: scores up to the 80th percentiles are labelled ‘Close to average’, scores between 81st and 90th percentiles are labelled ‘slightly raised (/slightly lowered)’, 91st and 95th percentile are labelled ‘high (/low)’ and scores higher than 95th percentile are labelled ‘very high (/very low)’. The labels in parentheses are used for the prosocial score which is reversed scored so that lower scores mean more problems.

## Results

### Representativeness

More participants from New Patterns had at least one parent with immigrant background (66%) compared with the general low-income population (59.3%).^27^ A higher percentage of participants from New Patterns also lived in a single parent household, that is, with mother alone or father alone (38.2%) compared with the general low-income population (36.1%). The parents’ educational levels were comparable to the general low-income population in Norway.^28^

### Descriptive statistics

Most participants had parents with secondary education or lower (86%) as their highest education, lived in rented residences or public housing (90%) and were unemployed (78%) (see table 1). The mean age of participating children and adolescents was 10.8 (SD=0.7), most had an immigrant background (66%) and most lived in a household with either a mother and father (38%) or a single mother (38%).

**Table 1:** Sociodemographic characteristics of participants at baseline

	N	%
Highest education level in the family	152	
Not finished primary school	24	16
Primary school	54	36
Secondary school	52	34
Higher education	22	14
Family housing	152	
Owns residence	15	10
Rents residence	86	56
Rents public housing	51	34
Parental employment status	152	
Unemployed	119	78
Employed	33	22
Children’s immigrant status	301	
Non-immigrant background	102	34
Immigrant background*	199	66
Child born in Norway	201	67
Children’s gender	301	
Female	162	54
Male	139	46
Children’s Age (M, (SD))	10.76	(3.74)
Children’s living arrangement	301	
Mother alone	113	38
Father alone	13	4
Mother and father	115	38
Lives with mother, visit father	50	17
Other (shared residence or lives with father and visit mother)	10	3

*Immigrant or born in Norway with one or two immigrant parents.

### Mental health problems by sex and immigrant background

Table 2 presents descriptive statistics and subgroup differences in SDQ scores reported by parents. The total sample mean was 10.7 (SD=6.7), with the highest subscale problem score being hyperactivity (M=4.5, SD=2.8). Most children were rated high on prosocial skills by their parents with a mean close to 9. Boys were rated as having significantly more symptoms of hyperactivity than girls (mean difference 0.97 (95% CI 0.07 to 1.9)). Boys were also rated as significantly less prosocial than girls (mean difference −0.81 (95% CI −1.3 to −0.3)). Children from a non-migrant background were rated as having significantly higher Total Difficulties Scale scores (mean difference 2.86 (95% CI 0.7 to 5)). Non-immigrant children were also rated as having more symptoms of emotional difficulties, conduct problems, hyperactivity/inattention problems and were also rated as less prosocial than their peers with immigrant backgrounds. A sensitivity check of parent-reported data revealed that there were minor differences between those with immigrant background who were born in Norway (M=9.5, SD=6.5) and those born outside of Norway (M=9.5, SD=5.8) on the Total SDQ difficulties score.

**Table 2:** Parent reported strengths and difficulties score, stratified by sex and migration background

	Total	Sex			Background		
	(1)	(2)	(3)	(4)	(5)	(6)	(7)
	Total (n=153)	Girls (n=61)	Boys (n=92)	Mean difference	Immigrant (n=87)	Non-immigrant (n=66)	Mean difference
	Mean (SD)	Mean (SD)	Mean (SD)	Mean (95 % CI)	Mean (SD)	Mean (SD)	Mean (95 % CI)
Total difficulties	10.74 (6.68)	9.84 (6.79)	11.34 (6.58)	1.50 (−0.67, 3.67)	9.51 (6.26)	12.36 (6.93)	2.86 (0.73, 4.98)†
Emotional symptoms	2.56 (2.36)	2.62 (2.58)	2.52 (2.23)	−0.10 (−0.89, 0.69)	2.18 (2.19)	3.06 (2.51)	0.88 (0.12, 1.64)*
Conduct problems	2.00 (1.96)	1.79 (1.98)	2.14 (1.94)	0.35 (−0.28, 0.99)	1.53 (1.63)	2.62 (2.18)	1.09 (0.46, 1.72)‡
Hyperactivity	4.48 (2.82)	3.90 (2.78)	4.87 (2.79)	0.97 (0.07, 1.87)*	4.08 (2.65)	5.02 (2.97)	0.93 (0.03, 1.84)*
Peer problems	1.69 (1.84)	1.52 (1.64)	1.80 (1.97)	0.28 (−0.30, 0.86)	1.71 (1.91)	1.67 (1.77)	−0.05 (−0.63, 0.54)
Prosocial	8.69 (1.78)	9.18 (1.31)	8.37 (1.97)	−0.81 (−1.33 to –0.29)†	8.99 (1.56)	8.30 (1.98)	−0.69 (−1.26 to –0.11)*
Impact	1.27 (2.39)	0.85 (1.71)	1.55 (2.72)	0.70 (−0.00, 1.40)	1.03 (2.61)	1.59 (2.05)	0.56 (−0.18, 1.29)

Column 1: Mean score parent reported SDQ. SD in parentheses. Column 2: mean score girls. SD in parentheses. Column 3: mean score for boys. SD in parentheses. Column 4: mean difference in SDQ scores between boys and girls with 95% CIs in brackets using t-test assuming unequal variance. Column 5: mean score for children with immigrant background (immigrant or born in Norway with one or two immigrant parents). SD in parentheses. Column 6: mean score for children with non-immigrant background (born in Norway with parents without immigrant background). SD in parentheses. Column 7: mean difference in SDQ scores between children with and without immigrant background with 95% CIs in brackets using t-test assuming unequal variance.

*p<0.05.

†p<0.01.

‡p<0.001.

SDQ, Strengths and Difficulties Questionnaire.

Self-reported SDQ scores are presented in table 3. The total sample mean for SDQ total difficulties was about 10 (SD=5.6), and the highest problem subscale was hyperactivity/inattention problems (M=3.9, SD=2.5). Boys rated themselves significantly less prosocial than girls (mean difference=−0.8 (95% CI −1.30 to –0.24). Non-immigrant children scored higher on all SDQ problem subscales (eg, the mean difference on the Total Difficulties Scale was 5.3 (95% CI 3.46 to 7.19)) compared with their peers with immigrant background. Non-immigrant children also scored significantly lower on the prosocial scale (−0.8 (95% CI−1.46 to –0.17)) and they perceived their symptoms of mental health problems as significantly more impairing, as indicated by a higher impact score (mean difference 1.1 (95% CI 0.13 to 2.02).

**Table 3:** Self-reported Strengths and Difficulties Score, stratified by sex and migration background

	Total	Gender			Background		
	(1)	(2)	(3)	(4)	(5)	(6)	(7)
	Total (n=148)	Girls (n=78)	Boys (n=70)	Mean difference	Immigrant (n=112)	Non-immigrant (n=36)	Mean difference
	Mean (SD)	Mean (SD)	Mean (SD)	Mean (95% CI)	Mean (SD)	Mean (SD)	Mean (95% CI)
Total difficulties	10.08 (5.57)	9.91 (5.60)	10.27 (5.56)	0.36 (−1.44, 2.16)	8.79 (5.14)	14.11 (4.91)	5.33 (3.46, 7.19)‡
Emotional symptoms	2.68 (2.22)	2.99 (2.40)	2.33 (1.96)	−0.66 (−1.36, 0.04)	2.17 (1.95)	4.25 (2.29)	2.08 (1.25, 2.91)‡
Conduct problems	1.52 (1.55)	1.50 (1.65)	1.54 (1.45)	0.04 (−0.46, 0.54)	1.35 (1.49)	2.06 (1.66)	0.71 (0.10, 1.31)*
Hyperactivity	3.93 (2.47)	3.60 (2.46)	4.30 (2.45)	0.70 (−0.10, 1.49)	3.53 (2.42)	5.19 (2.24)	1.67 (0.81, 2.53)‡
Peer problems	1.95 (1.58)	1.82 (1.54)	2.10 (1.62)	0.28 (−0.23, 0.79)	1.74 (1.51)	2.61 (1.63)	0.87 (0.27, 1.47)†
Prosocial	8.51 (1.66)	8.87 (1.31)	8.10 (1.90)	−0.77 (−1.30 to –0.24)†	8.71 (1.59)	7.89 (1.75)	−0.82 (−1.46 to –0.17)*
Impact	0.97 (2.08)	1.13 (2.16)	0.78 (1.99)	−0.35 (−1.02, 0.33)	0.70 (1.76)	1.78 (2.73)	1.08 (0.13, 2.02)*

Column 1: mean score self-reported SDQ. SD in parentheses. Column 2: mean score girls. SD in parentheses. Column 3: mean score for boys. SD in parentheses. Column 4: mean difference in SDQ scores between boys and girls with 95% CIs in brackets using t-test assuming unequal variance. Column 5: mean score for children with immigrant background (immigrant or born in Norway with one or two immigrant parents). SD in parentheses. Column 6: mean score for children with non-immigrant background (born in Norway with parents without immigrant background). SD in parentheses. Column 7: mean difference in SDQ scores between children with and without immigrant background with 95% CIs in brackets using t-test assuming unequal variance.

*p<0.05.

†p<0.01.

‡p<0.001.

SDQ, Strengths and Difficulties Questionnaire.

### Predictors of mental health differences from multiple linear regression

The results from the multiple linear regression analysis on predictors of SDQ scores can be seen in table 4.

**Table 4:** Results from adjusted multiple linear regression analyses predicting scores on the Strengths and Difficulties Questionnaire (SDQ)

	(1)	(2)	(3)	(4)	(5)	(6)	(7)
	Total difficulties b (SE)	Emotional symptoms b (SE)	Conduct problems b (SE)	Hyperactivity b (SE)	Peer problems b (SE)	Prosocial b (SE)	Impact b (SE)
Panel A: Parent-reported SDQ							
Girl	−1.130 (1.253)	0.216 (0.422)	−0.191 (0.344)	−0.828 (0.514)	−0.327 (0.311)	0.704 (0.280)*	−0.666 (0.386)
Age (mean centred)	0.369 (0.186)	0.200 (0.0622)†	0.0752 (0.0572)	−0.0389 (0.0698)	0.133 (0.0595)*	0.0336 (0.0502)	0.190 (0.0833)*
Immigrant background	−2.805 (1.254)*	−1.014 (0.431)*	−1.083 (0.369)†	−0.747 (0.500)	0.0390 (0.329)	0.558 (0.323)	−0.539 (0.411)
Time	−0.004 (0.007)	0.000 (0.003)	−0.001 (0.002)	−0.003 (0.003)	0.000 (0.002)	−0.001 (0.002)	0.001 (0.002)
Constant	14.20 (1.275)‡	3.520 (0.432)‡	3.042 (0.411)‡	5.464 (0.485)‡	2.171 (0.423)‡	8.259 (0.323)‡	2.302 (0.514)‡
Observations	153	153	153	153	153	153	153
Panel B: Self-reported SDQ							
Girl	−0.586 (0.870)	0.550 (0.317)	−0.0630 (0.236)	−0.753 (0.407)	−0.320 (0.244)	0.808 (0.253)†	0.268 (0.344)
Age (mean centred)	0.298 (0.189)	0.161 (0.0734)*	−0.00598 (0.0534)	0.0569 (0.0964)	0.0863 (0.0681)	−0.0497 (0.0617)	0.122 (0.0899)
Immigrant background	−5.214 (0.880)‡	−1.974 (0.385)‡	−0.705 (0.256)†	−1.681 (0.421)‡	−0.855 (0.304)†	0.850 (0.324)*	−1.017 (0.503)*
Time	−0.008 (0.006)	−0.001 (0.003)	−0.002 (0.002)	−0.004 (0.003)	−0.001 (0.002)	0.001 (0.002)	0.002 (0.003)
Constant	14.31 (1.201)‡	3.504 (0.491)‡	2.298 (0.282)‡	5.862 (0.601)‡	2.642 (0.412)‡	7.436 (0.445)‡	1.066 (0.627)
Observations	148	148	148	148	148	148	147

Immigrant background is a dummy variable indicating if the child grows up in an immigrant household. Age is a continuous variable measuring age in years at the time when SDQ is reported, this variable was mean centred in the analyses. Girl is a dummy variable indication if the child is a girl. Time is a continuous variable measuring the number of days since a family coordinator was assigned to the family. Robust SEs were clustered on the family level. The first line in each row presents the estimated coefficient, the second presents the standard errors (in parentheses).

*p<0.05.

†p<0.01.

‡p<0.001.

In the adjusted regression analyses, immigrant background was a significant predictor of fewer total difficulties (b=−2.8, SE=1.3), symptoms of emotional problems (b=1.01, se=0.4) and conduct problems (b=−1.08, se=0.4) in parent-reported data, and across all SDQ subscales in self-reported data (see Panel B in table 4). Older age was associated with more emotional symptoms (b=0.2, se=0.1), peer problems (b=0.04, se=0.1) and higher impact scores (b=0.19, se=0.1) in parent-reported data, and with more emotional symptoms (b=0.16, se=0.1) in self-reported data. Being a girl was associated with more prosocial behaviour in both parent-reported (b=0.7, se=0.3) and self-reported (b=0.8, se=0.3) SDQ data.

### Categorisation of scores according to UK 4-band categories

**Figure 1:**
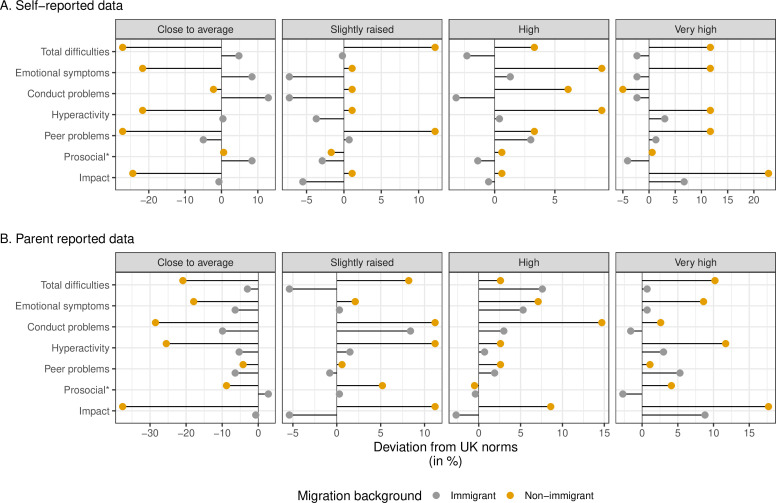
Self-reported and parent-reported scores on the Strengths and Difficulties Questionnaire for immigrant and non-immigrant participants as deviations from the UK 4-band solution. The figure illustrates the scores in the current sample as deviations from the expected distribution based on the UK 4-band solution.^27^ Category labels are printed on top of each facet in the plot. Negative values suggest that there are fewer than expected that score within a category based on UK norms, positive values indicate that more participants than expected score within that category whereas values of 0 suggest that scores correspond to distributions from the UK 4-band solution. Note that the scales of the x-axes are free.

To compare the distribution of scores in our current sample with UK norms, we created cut-points corresponding to the UK 4-band solution for self-report and parent-report for immigrant and non-immigrant participants (see figure 1). Figure 1 illustrates the deviation in percentages from the UK norms for each of the four bands (close to average, slightly raised, high and very high) by informant.

The self-reported SDQ scores (Panel A, figure 1) show that fewer than expected non-immigrant children (yellow circles) score close to average whereas more score in the slightly raised, high and very high categories for most problems measured by the SDQ. For prosocial behaviours, the pattern of results closely resembles the UK-norms. A different pattern emerged for children with immigrant backgrounds (grey circles). More children with migrant background than expected score near or in the close to average range, and fewer non-immigrant children obtain scores that are classified into the slightly raised, high and very high categories. Two exceptions were hyperactivity/inattention and impact, where more children than expected obtained scores in the very high category.

In parent reported data (Panel B, figure 1), the patterns of results were more similar for participants with and without immigrant background. Fewer children without immigrant background than expected were given scores in the close to average category, whereas more children obtained scores categorised as slightly raised, high or very high. The largest deviations from UK norms were obtained for non-immigrant participants (yellow circles) in the very high category, with two to three times as many non-immigrant children as expected scoring into this category for Total difficulties, emotional symptoms, hyperactivity/inattention, peer problems and impact. For children with immigrant background, more than expected obtained very high scores on impact, peer problems and hyperactivity.

## Discussion

The current study presents results from parent-reported and self-reported mental health baseline screening of child and adolescent participants from an intervention targeting low-income families in Norway. The results suggest that participants had many symptoms of mental health problems; older children had more symptoms of emotional and peer problems and higher impact scores as reported by their parents, and more self-reported emotional symptoms. A main finding in the current study was the existence of differences in mental health between participants with and without immigrant backgrounds, with native children having higher levels of problems across most domains.

When comparing the distribution of scores to the 4-band SDQ scoring solution based on UK norms, it appeared that non-immigrant participants obtained relatively higher scores, whereas immigrant participants obtained scores that were lower or more in alignment with the distribution of scores in the UK norm data. Importantly, previous studies have found lower SDQ scores among Nordic children and adolescents compared with peers in other (European) countries,^29^ so the scores obtained in the current study are higher than what has been reported previously. The finding of more symptoms of mental health problems in this sample can probably be attributed to the risk associated with low income and the challenging living conditions that characterises this sample.^14^

In general, participants obtained relatively high scores on parent reported total difficulties. In a previous review of the SDQ in Nordic countries, Obel *et al*^30^ report parent-reported SDQ scores in the range of 5.7–6.4 in children aged 7–9 years old from the Nordic countries. More recently, Gunnarsdóttir *et al*^31^ found a mean parent reported total difficulties score of 7.7 (SD=4.3) across a sample of more than 6000 Nordic 4–16 years. Moreover, the average self-reported Total Difficulties Scale scores in the current study were also relatively high, and comparable to scores previously reported by Obel *et al* for older Norwegian adolescents (13–15 years old).^30^ The pattern of findings related to age and gender was in line with previous findings using the SDQ^32^ although the scores from the current study were higher. The results are in general also aligned with normative gender-related and age-related changes in development of mental health problems.

Participants with immigrant background scored lower than previously reported while non-immigrants scored higher,^31^ and non-immigrants scored relatively high on UK norms. The finding of lower scores among participants with immigrant backgrounds where somewhat surprising as earlier studies in Norway and elsewhere have suggested that migration background can be a risk factor for mental health problems.^11–13 19^ In the broader literature, however, migration background has been associated with both lower and higher morbidity.^33 34^ The relationship between migration background and mental health outcomes may vary in line with different factors, including individual characteristics, SES, cultural adaptation and the circumstances surrounding the migration process. Overall, it is crucial to recognise the diversity within immigrant populations. One explanation for higher levels of mental health problems may be that some immigrants face significant challenges before they migrate, such as war, violence, discrimination or economic hardship in their home countries, potentially contributing to increased vulnerability to mental ill health.^35^ Moreover, some migrants may have limited social support in their new country, which can contribute to feelings of isolation and loneliness, also potentially increasing the risk of mental health problems.^36^

The low scores among migrant participants in the current study could be related to methodological challenges related to survey completion or to the functioning of the SDQ.^37^ Other studies have found parent reported SDQ scores to be functionally equivalent across immigration status.^38^ For the self-report version, one prior Norwegian study suggested that the total difficulties score worked well, but that there could be difficulties interpreting some of the subscale scores,^39^ but other studies have suggested that the subscales may work well among migrant participants.^40^ Also, more than 50% of the migrant sample were born in Norway, suggesting they should be able to adequately complete the SDQ. A sensitivity check of parent reported data revealed that there were minor differences between those with immigrant background who were born in Norway and those born outside of Norway on the Total SDQ difficulties score.

IAnother explanation for the discrepancies from findings could be that prior studies of migration-related differences have failed to adequately account for socioeconomic and other differences between participants with and without migration backgrounds.^41^ Prior studies may, therefore, have misattributed SES-related differences in mental health problems to migration background.^42^

The differences in mental health between the two samples could also be related to the levels of complexity surrounding the low-income situation for participants with and without migration background. Mental health problems in participants with migration backgrounds may mainly be linked to material deprivation related to the migration and resettling process.^43^ Deprivation may arise because migrants may not be able to access work for various reasons (eg, due to language difficulties, or their educational and/or professional qualifications not being acknowledged or recognised in their receiving society). Unemployment or underemployment may result in a household income too low to make ends meet for migrant families, causing stresses and strains on families that may result in increased mental health symptoms.^44^ Importantly, however, these stresses may be more transient for some and expected to eventually alleviate, thereby protecting the families from dysfunction resulting in less severe mental health problems in children with immigrant backgrounds. Studies do, for example, document that children of migrants achieve higher educational attainment and earnings as adults, in comparison to their native peers with similar SES.^45^ It may also be that migrants on average have better health, including mental health, compared with the native population, in line with the ‘health migrant effect’, although the evidence in support of this hypothesis appears to be inconclusive.^46^

For participants without migration background, however, the low-income situation may be more intricate. Beiser *et al*^42^ have documented that children with non-immigrant backgrounds that live in low-income households have more mental health problems than their peers with immigrant backgrounds, and that these problems are mostly mediated by single-parent status, less effective parenting practices, parental depression and family dysfunction. This is in line with previous studies that document how poverty may track indirectly over generations through poor quality parenting related to economict stress, parental mental health problems and family conflicts,^47^ in addition to more direct effects through transmission of earnings levels, welfare culture and human capital.^48–50^ Non-immigrant participants in poor families may, therefore, both inherit more risky family environments and genetic vulnerabilities^50^ and grow up within more generally impoverished surroundings accumulating their risks for developing mental health problems.

### Strengths and limitations

Among the strengths of the current study is the recruitment of a relatively large hard-to-reach sample of low-income families in Norway that includes both participants with and without migration background. Another strength is the use of a validated instrument to measure mental health problems. The findings from the current study should also be interpreted considering certain limitations; some of which regards the representativeness of the sample. Due to the recruitment procedure, the sample of participants may have more difficulties compared with a regular low-income sample of Norwegian families, and the results may, therefore, not readily generalise to this population. Finally, although the sample was large compared with other studies of low-income families in Norway, a larger sample would have resulted in more precise point estimates from the statistical comparisons.

## Conclusion

Child and adolescent participants participating in an intervention targeting low-income families in Norway had many symptoms of mental health problems at baseline, and participants with non-immigrant background had more severe problems. There is a need for more detailed assessments of the characteristics of families where children have significant mental health problems to better understand the mechanisms underlying the development of mental health problems in children who grow up in low-income families. This, in turn, could allow development of interventions that are better tailored to the different needs of the families and the children that grow up in these families.

## Supplementary Material

Author's manuscript

## Data Availability

There are legal and ethical restrictions on sharing the dataset used for the present manuscript. Access to the dataset require application and approval by Regional Committee for Medical and Health Research Ethics (REC) in Western Norway and the Norwegian Centre for Research Data (NSD). In addition, Norwegian Health research legislation and the Norwegian Ethics committees require explicit consent from participants in order to transfer health research data outside of Norway. In this specific case, ethics approval is also contingent on storing the research data on secure storage facilities located in our research institution.
