# Supplementary material for: Self-reported and parent-reported mental health in children from low-income families in Agder, Norway: results from baseline measurements of New Patterns project participants
Source: BMJ Open. 2023 Nov 27;13(11):e076400. doi: 10.1136/bmjopen-2023-076400 (PMC10685927; doi:10.1136/bmjopen-2023-076400)
Supplement: Author's manuscript [file bmjopen-2023-076400.draft_revisions.pdf]

BMJ Open is committed to open peer review. As part of this commitment we make the peer review history of every article we publish publicly available.

When an article is published we post the peer reviewers' comments and the authors' responses online. We also post the versions of the paper that were used during peer review. These are the versions that the peer review comments apply to.

The versions of the paper that follow are the versions that were submitted during the peer review process. They are not the versions of record or the final published versions. They should not be cited or distributed as the published version of this manuscript.

BMJ Open is an open access journal and the full, final, typeset and author-corrected version of record of the manuscript is available on our site with no access controls, subscription charges or pay-per-view fees (http://bmjopen.bmj.com).

If you have any questions on BMJ Open's open peer review process please email info.bmjopen@bmj.com

BMJ Open

Self- and parent-reported mental health in children from low-income families in Agder, Norway: Results from baseline measurements of New Patterns project participants

Journal:	*BMJ Open*
Manuscript ID	bmjopen-2023-076400
Article Type:	Original research
Date Submitted by the Author:	06-Jun-2023
Complete List of Authors:	Bøe, Tormod; University of Bergen, Department of Psychosocial Science; NORCE Norwegian Research Centre AS Forskningsområde Helse, Regional Centre for Child and Youth Mental Health and Child Welfare<br>Ostojic, Helene Angelica; Vestre Viken HF, Drammen District Psychiatric Center, Mental Health and Substance Abuse; University of Bergen, Department of Psychosocial Science<br>Haraldstad, Kristin; University of Agder, Dep of Health and Nursing Sciences, Faculty of health and Sport Sciences, University of Agder, Norway<br>Abildsnes, Eirik; University of Agder, Dep of Psychosocial Health<br>Wilson, Philip; University of Aberdeen, Centre for Rural Health<br>Vigsnes, Kristine; Kristiansand Kommune<br>Mølland, Eirin; University of Agder, School of Business and Law; NORCE Norwegian Research Centre AS Forskningsomrade Samfunn
Keywords:	SOCIAL MEDICINE, Health Equity, MENTAL HEALTH

SCHOLARONE™
Manuscripts

I, the Submitting Author has the right to grant and does grant on behalf of all authors of the Work (as defined in the below author licence), an exclusive licence and/or a non-exclusive licence for contributions from authors who are: i) UK Crown employees; ii) where BMJ has agreed a CC-BY licence shall apply, and/or iii) in accordance with the terms applicable for US Federal Government officers or employees acting as part of their official duties; on a worldwide, perpetual, irrevocable, royalty-free basis to BMJ Publishing Group Ltd ("BMJ") its licensees and where the relevant Journal is co-owned by BMJ to the co-owners of the Journal, to publish the Work in this journal and any other BMJ products and to exploit all rights, as set out in our licence.

The Submitting Author accepts and understands that any supply made under these terms is made by BMJ to the Submitting Author unless you are acting as an employee on behalf of your employer or a postgraduate student of an affiliated institution which is paying any applicable article publishing charge ("APC") for Open Access articles. Where the Submitting Author wishes to make the Work available on an Open Access basis (and intends to pay the relevant APC), the terms of reuse of such Open Access shall be governed by a Creative Commons licence – details of these licences and which Creative Commons licence will apply to this Work are set out in our licence referred to above.

Other than as permitted in any relevant BMJ Author's Self Archiving Policies, I confirm this Work has not been accepted for publication elsewhere, is not being considered for publication elsewhere and does not duplicate material already published. I confirm all authors consent to publication of this Work and authorise the granting of this licence.

For peer review only

Self- and parent-reported mental health in children from low-income families in Agder, Norway: Results from baseline measurements of New Patterns project participants

Tormod Bøe[a,b] *Helene Angelica Ostojic[a,c], Kristin Haraldstad[d], Eirik Abildsnes[e], Philip Wilson[f], Kristine Vigsnes[g] and Eirin Mølland[h,i]

[a] Department of Psychosocial Science, Faculty of Psychology, University of Bergen, Bergen, Norway,

[b] Regional Centre for Child and Youth Mental Health and Child Welfare, NORCE Norwegian Research Centre, Bergen, Norway

[c] Drammen District Psychiatric Center, Mental Health and Substance Abuse, Vestre Viken HC, Drammen, Norway

[d] Dep of Health and Nursing Sciences, Faculty of health and Sport Sciences, University of Agder, Norway

[e] Dep of Psychosocial Health, Faculty of Health and Sport Sciences, University of Agder, Norway

[f] Centre for Rural Health, Institute of Applied Health Sciences, University of Aberdeen, Scotland

[g] Kristiansand Municipality, Kristiansand, Norway

[h] School of Business and Law, University of Agder, Kristiansand, Norway

[i] NORCE Norwegian Research Centre, Bergen, Norway

*Corresponding author: Tormod Bøe, tormod.boe@uib.no

For peer review only

Key messages:

- Children and young people in this study of low-income families in Norway reported high levels of mental health problems.
- These problems appeared across different dimensions such as emotional-, peer-, and conduct problems, hyperactivity-/inattention, and lower prosocial skills. There was also evidence of corresponding functional impairment.
- The problems were more extensive among ethnic Norwegian participants than among peers with immigrant backgrounds.
- The study contributes information about within-sample-heterogeneity among children in low-income families which may be important to consider when designing interventions targeting these groups of children.

For peer review only

Abstract

Background. Poverty may pose risks to child and adolescent mental health, but few studies have reported on this association among children and adolescents in low-income families in Norway. **Methods.** Based on a sample participating in an intervention for low-income families in Norway, we report data from the survey administered at the start of the intervention. Mental health problems were measured using the Strengths and Difficulties Questionnaire (SDQ; self-report (SR) n = 148; parent/proxy-report (PR) n = 153, mean age = 10.8). Demographic and family characteristics were obtained from parent reported data. Results are presented by gender and migration background. Regression analysis was used to investigate the relative contribution of background factors to mental health symptoms. The distribution of scores is compared to UK norms.

Results. Participants reported relatively high scores on the SDQ Total Difficulties Scale (PR mean = 10.7; SR mean = 10.1). Participants with non-immigrant backgrounds scored considerably higher on the Total Difficulties Scale (PR mean difference = 2.9; SR 5.3) and on most other domains measured with the SDQ compared to their peers with immigration backgrounds. Participants generally scored higher than or equal to UK norms.

Conclusion. Participants in the current study had many symptoms of mental health problems, with large differences between those with and without a migrant background. Interventions for low-income families should be based on detailed knowledge about differences in family risks, resources and needs.

For peer review only

Introduction

Mental health problems in children and adolescents are common. Up to 20% of children and young adolescents have a mental disorder [1], and the prevalence may be rising, for girls in particular [2]. Mental health problems negatively impact young people's health and well-being, as well as their future educational prospects, employment and earnings [3]. Mental health problems also account for a substantial and increasing world-wide burden of ill-health [4].

Child and adolescent mental health problems are distributed unequally with regards to household income: More children and adolescents in families with low income have mental health problems compared to more affluent peers [5–7]. Children and adolescents with low socioeconomic status, (SES) are two to three times more likely to develop mental health problems than their peers with high SES [5,8]. Research suggests low income mainly influences children and adolescents indirectly, through constraining caregivers' abilities to deploy resources towards their development, and through increased family stress with detrimental consequences for parental mental health and parenting practices [9]. There may also be direct influences through toxic effects of poverty-related stress on the brains of children and adolescents, potentially influencing structure and function in brain areas involved in memory, emotion regulation, and executive function, with implications for mental health and cognitive functioning [10].

Migration background may also be related to elevated mental health problems in children and adolescents [11]. It was, for example, found that second-generation migrant children had higher rates of depression, anxiety and post-traumatic stress disorders compared to their native counterparts [12]. This is particularly relevant to the current study, as migrant families and their children make up a large portion of the low-income population in Norway [13].

The current study is based on data from *New Patterns*, an intervention study conducted in a sample of families with children younger than 17 purposefully recruited based on having household incomes below the low-income threshold in Norway (i.e. ≤ 60% of the equivalized population median income) as well as having longstanding needs for welfare services [14]. Families that are included in

For peer review only

New Patterns receive a family coordinator who is responsible for follow-up of all family members for a period of five years and coordinate services that are involved with the family. Norwegian register- and community-based observational studies have shown that children and adolescents from households with lower income and low socioeconomic status have a higher frequency of mental disorders relative to higher income peers [15]. Many children and adolescents with mental health problems nevertheless go undetected [16]. Furthermore, children from low-income families are less likely to participate in research and community programs compared to more affluent peers [17]. Therefore, register-based studies should be complemented by epidemiological studies directly targeting these low-income hard-to-reach samples to more fully capture the mental health status of children and adolescents who live in low-income families.

The aim of the study is to present results from the baseline mental health screening completed by participants in the New Patterns project. In presenting these data, we will provide results separately by informant; for younger children, their parent or a proxy completed the Strengths and Difficulties Questionnaire (SDQ), but older children completed it themselves. We also stratify our results by sex as differences in the trajectories of mental health problems among boys and girls are widely reported [18]. Finally, we present results separately for children and adolescents with and without immigrant background since previous studies have shown that ethnic minority children have higher mean SDQ scores compared to majority population samples in Norway [19].

Methods

Participants and procedures

The *New Patterns* project targets families with children aged 0-17 living in Norway with low household income and additional challenges requiring longstanding need for services [14]. Families were referred to the intervention from different service sectors within the municipalities (i.e., kindergarten, school, public health clinics, general practitioners, the Norwegian Labour and Welfare Administration (NAV), child protection services and mental health services). Each referral was subsequently discussed in a multidisciplinary intake-team who selected the families that were

For peer review only

considered eligible to participate based on their needs for coordinated longer-term follow-up. The study is conducted according to recommendations from the Norwegian Data Protection Services (record numbers 27435) and approved by the regional ethics committee of western Norway (approval number 249507).

Our sample consists of the baseline self-reported (number of participants (n) = 148, age mean (M) = 13.55, standard deviation (SD) = 1.95) and parent-reported (n = 153, age M = 8.07, SD = 3.01) SDQ questionnaires for children participating in the New Patterns project." The sample was restricted to reports that were gathered within 360 days after enrolment and for children in the age-range where Strengths and Difficulties Questionnaire (SDQ is validated (4-17 in the current study). The median time in the project when the SDQ was reported was 82 days. Twelve children were included in both the parent- and self-reported data.

Instruments

Mental health problems

Mental health was assessed with parent- and self-report versions of the extended Norwegian translation of the Strengths and Difficulties Questionnaire (SDQ) [20]. The psychometric properties of the SDQ have been well-documented [21,22]. The Norwegian translations of the SDQ have shown strong psychometric properties for all informant versions (abstract in English available at the PSYKTESTBARN website: shorturl.at/DUVY2), and have also received support for administration to 18-19-year-olds [23]. The SDQ contains 25 items, each of which can be answered using: "certainly true", "somewhat true", or "not true". They can be summed into five subscales each containing five items with a potential range of scores from 0 to 10: *conduct problems, hyperactivity/inattention, emotional problems, peer relationship problems,* and *prosocial behaviour*. The first four subscales can be added together as a *Total Difficulties Scale*, with a potential range from 0 to 40. The extended SDQ includes an impact supplement with items measuring functional impairment regarding chronicity, distress, social impairment, and burden to others [20].

For peer review only

Sociodemographic variables

Background characteristics were measured when the family entered the New Patterns project. *Parental education* was assessed as the highest level of education completed by the mother or father. If at least one of the parents in the household was employed at the time of entering the project, we defined parental employment status to be *employed*. Children who were born in Norway with one or two parents who had immigrated to Norway or children who themselves had immigrated to Norway were defined as having an *immigrant background.* Children born in Norway whose parents did not have immigrant background were defined as having a *non-immigrant background.* We used information about year of birth and the date of answering the SDQ questionnaire to compute approximately the age at which the SDQ questionnaire was completed.

Statistical analyses

Mean differences with 95% confidence intervals (CI) in SDQ-scores between boys and girls, and between children with immigrant and non-immigrant background were analysed using *t*-tests assuming unequal variance. Self-reported and parent-reported data were analysed separately. We then proceeded to estimate the following regression model:

$$y_i = \beta_0 + \beta_1 girl_i + \beta_2 age_i + \beta_3 Immigrant_i + \beta_4 Time_i + \epsilon_i$$

Where y_i is the outcome variable; SDQ Total Difficulties Scale score and the five-subscales. The variable *girl* is a dummy variable indicating if the individual is a girl, *age* is a continuous variable measuring age in years at the time when SDQ was reported. This variable was mean centred in the analyses ($age_{centered}$ = age – mean(age)) to ease interpretability of the regression coefficients. *Immigrant* is a dummy variable indicating if the child has immigrant background. *Time* is a continuous variable measuring the number of days since a family coordinator was assigned to the family. Robust standard errors were clustered on the family level. Stata version 17 was used for all analyses [24] whereas the R-package *ggplot2* [25] was used for making figures.

For peer review only - http://bmjopen.bmj.com/site/about/guidelines.xhtml

To compare the distribution of scores in our current sample with UK norms, we created cut-points corresponding to the UK 4-band solution [26]. These cut-points are based on percentile distributions in a large UK sample and use the categories: scores up to the 80th percentiles are labelled “Close to average”, scores between 81st-90th percentiles are labelled “Slightly raised (/Slightly lowered)”, 91st-95th percentile are labelled “High (/Low)” and scores higher than 95th percentile are labelled “Very high (/Very low)”. The labels in parenthesis are used for the prosocial score which is reversed scored so that lower scores mean more problems.

Results

Representativeness

More participants from *New Patterns* had a at least one parent with immigrant background (66%) compared to the general low-income population (59.3%) [27]. A higher percentage of participants from *New Patterns* also lived in a single parent household, i.e., with mother alone or father alone (38.2%) compared to the general low-income population (36.1%). The parents’ educational levels were comparable to the general low-income population in Norway [28].

Descriptive statistics

Most participants had parents with secondary education or lower (86%) as their highest education, lived in rented residences or public housing (90 %) and were unemployed (78%), see Table 1. The mean age of participating children and adolescents was 10.8 (SD = .7), most had an immigrant background (66%) and most lived in a household with either a mother and father (38%) or a single mother (38%).

[Table 1 near here]

Mental health problems by sex and immigrant background

Table 2 presents descriptive statistics and subgroup differences in SDQ scores reported by parents. The total sample mean was 10.7 (SD = 6.7), with the highest subscale problem score being hyperactivity (M = 4.5, SD = 2.8). Most children were rated high on prosocial skills by their parents

For peer review only

with a mean close to 9. Boys were rated as having significantly more symptoms of hyperactivity than girls (mean difference .97 [95% CI = .07, 1.9]). Boys were also rated as significantly less prosocial than girls (mean difference -.81, [95% CI = -1.3, -.3]). Children from a non-migrant background were rated as having significantly higher Total Difficulties Scale scores (mean difference 2.86, [95% CI = .7, 5]). Non-immigrant children were also rated as having more symptoms of emotional difficulties, conduct problems, hyperactivity/inattention problems and were also rated as less prosocial than their peers with immigrant backgrounds. A sensitivity check of parent reported data revealed that there were minor differences between those with immigrant background who were born in Norway (*M* = 9.5, *SD* = 6.5) and those born outside of Norway (*M* = 9.5, *SD* = 5.8) on the Total SDQ difficulties score.

[Table 2 near here]

Self-reported SDQ scores are presented in Table 3. The total sample mean for SDQ total difficulties was about 10 (SD = 5.6), and the highest problem subscale was hyperactivity/inattention problems (M = 3.9, SD = 2.5). Boys rated themselves significantly less prosocial than girls (mean difference = -.8 [95% CI = -1.30, -0.24]. Non-immigrant children scored higher on all SDQ problem subscales (e.g., the mean difference on the Total Difficulties Scale was 5.3 [95% CI = 3.46, 7.19]) compared to their peers with immigrant background. Non-immigrant children also scored significantly lower on the prosocial scale (-.8 [95% CI = [-1.46, -0.17]) and they perceived their symptoms of mental health problems as significantly more impairing, as indicated by a higher impact score (mean difference = 1.1, [95% CI = 0.13, 2.02].

[Table 3 near here]

Predictors of mental health differences from multiple linear regression

The results from the multiple linear regression analysis on predictors of SDQ scores can be seen in Table 4.

[Table 4 near here]

For peer review only - http://bmjopen.bmj.com/site/about/guidelines.xhtml

In the adjusted regression analyses, immigrant background was a significant predictor of fewer total difficulties (*b* = -2.8, *se* = 1.3), symptoms of emotional problems (*b* = 1.01, *se* = 0.4) and conduct problems (*b* = -1.08, *se* = 0.4) in parent-reported data, and across all SDQ subscales in self-reported data (see Panel B in Table 4). Older age was associated with more emotional symptoms (*b* = 0.2, *se* = 0.1), peer problems (*b* = 0.04, *se* = 0.1) and higher impact scores (*b* = 0.19, *se* = 0.1) in parent-reported data, and with more emotional symptoms (*b* = 0.16, *se* = 0.1) in self-reported data. Being a girl was associated with more prosocial behaviour in both parent- (*b* = 0.7, *se* = 0.3) and self-reported (*b* = 0.8, *se* = 0.3) SDQ data.

Categorization of scores according to UK 4-band categories

To compare the distribution of scores in our current sample with UK norms, we created cut-points corresponding to the UK 4-band solution for self- and parent report for immigrant and non-immigrant participants (see Figure 1). Figure 1 illustrate the deviation in percentages from the UK norms for each of the 4-bands (*close to average*, *slightly raised*, *high* and *very high*) by informant.

[Figure 1 near here]

The self-reported SDQ scores (Panel A, Figure 1) show that fewer than expected non-immigrant children (yellow circles) score *close to average* whereas more score in the *slightly raised*, *high* and *very high* categories for most problems measured by the SDQ. For prosocial behaviours, the pattern of results closely resembles the UK-norms. A different pattern emerged for children with immigrant backgrounds (grey circles). More children with migrant background than expected score near or in the *close to average range*, and fewer non-immigrant children obtain scores that are classified into the *slightly raised*, *high* and *very high* categories. Two exceptions were hyperactivity/inattention and impact, where more children than expected obtained scores in the *very high* category.

For peer review only

In parent reported data (Panel B, Figure 1) the patters of results were more similar for participants with and without immigrant background. Fewer children without immigrant background than expected were given scores in the close to average category, whereas more children obtained scores categorised as *slightly raised*, *high* or *very high*. The largest deviations from UK norms were obtained for non-immigrant participants (yellow circles) in the Very high category, with two-three times as many non-immigrant children as expected scoring into this category for Total difficulties, emotional symptoms, hyperactivity/inattention, peer problems and impact. For children with immigrant background, more than expected obtained very high scores on impact, peer problems and hyperactivity.

Discussion

The current study presents results from parent- and self-reported mental health baseline screening of child and adolescent participants from an intervention targeting low-income families in Norway. The results suggest that participants had many symptoms of mental health problems; older children had more symptoms of emotional and peer problems and higher impact scores as reported by their parents, and more self-reported emotional symptoms. A main finding in the current study was the existence of differences in mental health between participants with and without immigrant backgrounds, with native children having higher levels of problems across most domains.

When comparing the distribution of scores to the 4-band SDQ scoring solution based on UK norms, it appeared that non-immigrant participants obtained relatively higher scores, whereas immigrant participants obtained scores that were lower or more in alignment with the distribution of scores in the UK norm data. Importantly, previous studies have found lower SDQ scores among Nordic children and adolescents compared to peers in other (European) countries [29], so the scores obtained in the current study are higher than what has been reported previously. The finding of more symptoms of mental health problems in this sample can probably be attributed to the risk associated with low income and the challenging living conditions that characterises this sample [14].

For peer review only

In general, participants obtained relatively high scores on parent reported total difficulties. In a previous review of the SDQ in Nordic countries, Obel et al. [30] report parent-reported SDQ scores in the range of 5.7-6.4 in 7- to 9-year-old children from the Nordic countries. More recently, Gunnardsdottir [31] found a mean parent reported total difficulties score of 7.7 (SD = 4.3) across a sample of more than 6000 Nordic 4-16-year-olds. Moreover, the average self-reported Total Difficulties Scale scores in the current study were also relatively high, and comparable to scores previously reported by Obel et al. for older Norwegian adolescents (13-15-year-olds) [30]. The pattern of findings related to age and gender was in line with previous findings using the SDQ [32] although the scores from the current study were higher. The results are in general also aligned with normative gender- and age-related changes in development of mental health problems. Participants with immigrant background scored lower than previously reported while non-immigrants scored higher [30], and non-immigrants scored relatively high on UK norms. The finding of lower scores among participants with immigrant backgrounds where somewhat surprising as earlier studies in Norway and elsewhere have suggested that migration background can be a risk factor for mental health problems [11–13,19]. In the broader literature, however, migration background has been associated with both lower and higher morbidity [33,34]. The relationship between migration background and mental health outcomes may vary in line with different factors, including individual characteristics, socioeconomic status, cultural adaptation, and the circumstances surrounding the migration process. Overall, it is crucial to recognize the diversity within immigrant populations. One explanation for higher levels of mental health problems may be that some immigrants face significant challenges before they migrate, such as war, violence, discrimination, or economic hardship in their home countries, potentially contributing to increased vulnerability to mental ill-health [35]. Moreover, some migrants may have limited social support in their new country, which can contribute to feelings of isolation and loneliness, also potentially increasing the risk of mental health problems [36].

For peer review only

The low scores among migrant participants in the current study could be related to methodological challenges related to survey completion or to the functioning of the SDQ [37]. Other studies have found parent reported SDQ scores to be functionally equivalent across immigration status [38]. For the self-report version, one prior Norwegian study suggested that the total difficulties score worked well, but that there could be difficulties interpreting some of the subscale scores [39], but other studies have suggested that the subscales may work well among migrant participants [40]. Also, more than 50% of the migrant sample were born in Norway, suggesting they should be able to adequately complete the SDQ. A sensitivity check of parent reported data revealed that there were minor differences between those with immigrant background who were born in Norway and those born outside of Norway on the Total SDQ difficulties score.

Another explanation for the discrepancies from findings could be that prior studies of migration-related differences have failed to adequately account for socioeconomic- and other differences between participants with and without migration backgrounds [41]. Prior studies may therefore have misattributed SES-related differences in mental health problems to migration background [42].

The differences in mental health between the two samples could also be related to the levels of complexity surrounding the low-income situation for participants with and without migration background. Mental health problems in participants with migration backgrounds may mainly be linked to material deprivation related to the migration and resettling process [43]. Deprivation may arise because migrants may not be able to access work for various reasons (e.g., due to language difficulties, or their educational and/or professional qualifications not being acknowledged or recognized in their receiving society). Unemployment or underemployment may result in a household income too low to make ends meet for migrant families, causing stresses and strains on families that may result in increased mental health symptoms [44]. Importantly however, these stresses may be more transient for some and expected to eventually alleviate, thereby protecting the families from dysfunction resulting in less severe mental health problems in children with immigrant

For peer review only

backgrounds. Studies do for example document that children of migrants achieve higher educational attainment and earnings as adults, in comparison to their native peers with similar socioeconomic status [45]. It may also be that migrants on average have better health, including mental health, compared to the native population, in line with the “health migrant effect”, although the evidence in support of this hypothesis appears to be inconclusive [46].

For participants without migration background, however, the low-income situation may be more intricate. Beiser et al. [43] have documented that children with non-immigrant backgrounds that live in low-income households have more mental health problems than their peers with immigrant backgrounds, and that these problems are mostly mediated by single-parent status, less effective parenting practices, parental depression and family dysfunction. This is in line with previous studies that document how poverty may track indirectly over generations through poor quality parenting related to economic stress, parental mental health problems and family conflicts [47], in addition to more direct effects through transmission of earnings levels, welfare culture and human capital [48–50]. Non-immigrant participants in poor families may therefore both inherit more risky family environments and genetic vulnerabilities [51] and grow up with in more generally impoverished surroundings accumulating their risks for developing mental health problems.

Strengths and limitations

Among the strengths of the current study is the recruitment of a relatively large hard-to-reach sample of low-income families in Norway that includes both participants with and without migration background. Another strength is the use of a validated instrument to measure mental health problems. The findings from the current study should also been interpreted considering certain limitations; some of which regards the representativeness of the sample. Due to the recruitment procedure, the sample of participants may have more difficulties compared to a regular low-income sample of Norwegian families, and the results may therefore not readily generalize to this population. Finally, although the sample was large compared to other studies of low-income families

For peer review only

in Norway, a larger sample would have resulted in more precise point estimates from the statistical comparisons.

Conclusion

Child and adolescent participants participating in an intervention targeting low-income families in Norway had many symptoms of mental health problems at baseline, and participants with non-immigrant background had more severe problems. There is a need for more detailed assessments of the characteristics of families where children have significant mental health problems to better understand the mechanisms underlying the development of mental health problem sin children who grow up in low-income families. This in turn could allow development of interventions that are better tailored to the different needs of the families and the children that grow up in these families.

For peer review only

References

1 Polanczyk GV, Salum GA, Sugaya LS, *et al.* Annual research review: a meta-analysis of the worldwide prevalence of mental disorders in children and adolescents. *J Child Psychol Psychiatry Allied Discip* 2015;**56**:345–65. doi:10/gdsndp

2 Collishaw S. Annual Research Review: Secular trends in child and adolescent mental health. *J Child Psychol Psychiatry* 2015;**56**:370–93. doi:10/f68wxn

3 Evensen M, Lyngstad TH, Melkevik O, *et al.* Adolescent mental health and earnings inequalities in adulthood: evidence from the Young-HUNT Study. *J Epidemiol Community Health* 2017;**71**:201–6. doi:10/f9kpvg

4 Murray CJL, Vos T, Lozano R, *et al.* Disability-adjusted life years (DALYs) for 291 diseases and injuries in 21 regions, 1990–2010: a systematic analysis for the Global Burden of Disease Study 2010. *The Lancet* 2012;**380**:2197–223. doi:10/f2f8rw

5 Reiss F. Socioeconomic inequalities and mental health problems in children and adolescents: a systematic review. *Soc Sci Med* 2013;**90**:24–31. doi:10/f43jnn

6 Barry SJE, Marryat L, Thompson L, *et al.* Mapping area variability in social and behavioural difficulties among Glasgow pre-schoolers: linkage of a survey of pre-school staff with routine monitoring data: Mapping area variability. *Child Care Health Dev* 2015;**41**:853–64. doi:10/f77dr6

7 Reiss F, Meyrose A-K, Otto C, *et al.* Socioeconomic status, stressful life situations and mental health problems in children and adolescents: Results of the German BELLA cohort-study. *PLOS ONE* 2019;**14**:e0213700. doi:10/gfz4x8

8 Marryat L, Thompson L, Minnis H, *et al.* Primary schools and the amplification of social differences in child mental health: a population-based cohort study. *J Epidemiol Community Health* 2018;**72**:27–33. doi:10/ggk725

9 Gershoff ET, Aber JL, Raver CC, *et al.* Income is not enough: incorporating material hardship into models of income associations with parenting and child development. *Child Dev* 2007;**78**:70–95. doi:10/gw5

10 Johnson SB, Riis JL, Noble KG. State of the Art Review: Poverty and the Developing Brain. *Pediatrics* 2016;:peds.2015-3075. doi:10/f8gmmh

11 Belhadj Kouider E, Koglin U, Petermann F. Emotional and behavioral problems in migrant children and adolescents in Europe: a systematic review. *Eur Child Adolesc Psychiatry* 2014;**23**:373–91. doi:10/gmbft5

12 Ceri V, Özlü-Erkilic Z, Özer Ü, *et al.* Mental health problems of second generation children and adolescents with migration background. *Int J Psychiatry Clin Pract* 2017;**21**:142–7. doi:10/gr8sp3

13 Povlsen L, Regber S, Fosse E, *et al.* Economic poverty among children and adolescents in the Nordic countries. *Scand J Public Health* 2018;**46**:30–7. doi:10/gc8vg2

14 Mølland E, Vigsnes KL, Bøe T, *et al.* The New Patterns study: coordinated measures to combat child poverty. *Scand J Public Health* 2020;:1403494820956452. doi:10/ghbm4k

15 Kinge JM, Øverland S, Flatø M, *et al.* Parental income and mental disorders in children and adolescents: prospective register-based study. *Int J Epidemiol* Published Online First: 11 May 2021. doi:10/gj7skq

16 Heiervang E, Stormark KM, Lundervold AJ, *et al.* Psychiatric disorders in Norwegian 8- to 10-year-

For peer review only

olds. *J Am Acad Child Adolesc Psychiatry* 2007;**46**:438–47. doi:10/d8kr3v

17 Drummond J, Schnirer L, So S, *et al.* The protocol for the Families First Edmonton trial (FFE): a randomized community-based trial to compare four service integration approaches for families with low-income. *BMC Health Serv Res* 2014;**14**:223. doi:10/f56s6h

18 Patalay P, Fitzsimons E. Development and predictors of mental ill-health and wellbeing from childhood to adolescence. *Soc Psychiatry Psychiatr Epidemiol* 2018;**53**:1311–23. doi:10/gfkkz2

19 Sagatun Å, Lien L, Søgaard AJ, *et al.* Ethnic Norwegian and ethnic minority adolescents in Oslo, Norway: A longitudinal study comparing changes in mental health. *Soc Psychiatry Psychiatr Epidemiol* 2008;**43**:87–95. doi:10/c7g4dt

20 Goodman R. The extended version of the strengths and difficulties questionnaire as a guide to child. *J Child Psychol Psychiatry Allied Discip* 1999;**40**:791. doi:10/dk7tcs

21 Muris P, Meesters C, Van den Berg F. The strengths and difficulties questionnaire (SDQ) further evidence for its reliability and validity in a community sample of dutch children and adolescents. *Eur Child Adolesc Psychiatry* 2003;**12**:1–8. doi:10/fc65c3

22 Ortuño-sierra J, Fonseca-pedrero E, Aritio-solana R, *et al.* New evidence of factor structure and measurement invariance of the sdq across five european nations. *Eur Child Adolesc Psychiatry* 2015;**24**:1523–34. doi:10/f7z84r

23 Bøe T, Hysing M, Skogen JC, *et al.* The strengths and difficulties questionnaire (SDQ): factor structure and gender equivalence in Norwegian adolescents. *PLoS One* 2016;**11**:1–15. doi:10/f9v8vm

24 StataCorp. Stata Statistical Software. 2022.

25 Wickham H. *ggplot2: Elegant Graphics for Data Analysis*. Springer-Verlag New York 2016. https://ggplot2.tidyverse.org

26 Youthinmind.org. Scoring the Strengths & Difficulties Questionnaire for age 4-17 or 18+. Scoring SDQ. 2016.https://www.sdqinfo.org/py/sdqinfo/c0.py (accessed 9 Oct 2022).

27 Normann TM, Epland J. Fremdeles 115 000 barn med vedvarende lavinntekt i 2020. SSB. 2022.https://www.ssb.no/inntekt-og-forbruk/inntekt-og-formue/statistikk/inntekts-og-formuesstatistikk-for-husholdninger/artikler/fremdeles-115-000-barn-med-vedvarende-lavinntekt-i-2020 (accessed 20 Sep 2022).

28 Bufdir. Barnefattigdom. 2022.https://bufdir.no/Statistikk_og_analyse/Barnefattigdom/#/4204 (accessed 20 Sep 2022).

29 Elberling H, Linneberg A, Olsen EM, *et al.* The prevalence of sdq-measured mental health problems at age 5-7 years and identification of predictors from birth to preschool age in a danish birth cohort: the copenhagen child cohort 2000. *Eur Child Adolesc Psychiatry* 2010;**19**:725–35. doi:10/bczc5d

30 Obel C, Heiervang E, Rodriguez A, *et al.* The Strengths and Difficulties questionnaire in the Nordic countries. *Eur Child Adolesc Psychiatry Suppl* 2004;**13**:II32-9. doi:10/d4bhcq

31 Gunnarsdottir H, Hensing G, Povlsen L, *et al.* Relative deprivation in the Nordic countries-child mental health problems in relation to parental financial stress. *Eur J Public Health* 2016;**26**:277–82. doi:10/f3m7dz

32 Niclasen J, Teasdale TW, Andersen AMN, *et al.* Psychometric properties of the danish strength and difficulties questionnaire: the sdq assessed for more than 70,000 raters in four different cohorts. *PLoS One* 2012;**7**:e32025. doi:10/ggmjt3

For peer review only

33 Sam DL, Vedder P, Liebkind K, *et al.* Immigration, acculturation and the paradox of adaptation in Europe. *Eur J Dev Psychol* 2008;**5**:138–58. doi:10/c37r49

34 Schwartz SJ, Unger JB, Zamboanga BL, *et al.* Rethinking the concept of acculturation: Implications for theory and research. *Am Psychol* 2010;**65**:237–51. doi:10/c97xbh

35 Lebano A, Hamed S, Bradby H, *et al.* Migrants' and refugees' health status and healthcare in Europe: a scoping literature review. *BMC Public Health* 2020;**20**:1039. doi:10/gjfj68

36 Gleeson C, Frost R, Sherwood L, *et al.* Post-migration factors and mental health outcomes in asylum-seeking and refugee populations: a systematic review. *Eur J Psychotraumatology* 2020;**11**:1793567. doi:10/gm6dtn

37 Maldonado BN, Chandna J, Gladstone M. A systematic review of tools used to screen and assess for externalising behavior symptoms in low and middle income settings. *Glob Ment Health* 2019;**6**:Article e13. doi:10/gmwdx6

38 Runge RA, Soellner R. Measuring children's emotional and behavioural problems: are SDQ parent reports from native and immigrant parents comparable? *Child Adolesc Psychiatry Ment Health* 2019;**13**:1–15. doi:10/gqzdsj

39 Richter J, Sagatun $\backslash$AAse, Heyerdahl S, *et al.* The Strengths and Difficulties Questionnaire (SDQ) - Self-Report. an Analysis of Its Structure in a Multiethnic Urban Adolescent Sample. *J Child Psychol Psychiatry Allied Discip* 2011;**52**:1002–11. doi:10/d7wbhg

40 Stevanovic D, Urbán R, Atilola O, *et al.* Does the Strengths and Difficulties Questionnaire – self report yield invariant measurements across different nations? Data from the International Child Mental Health Study Group. *Epidemiol Psychiatr Sci* 2015;**24**:323–34. doi:10/gd59p2

41 Stevens GWJM, Buyukcan-Tetik A, Maes M, *et al.* Examining Socioeconomic Disparities in Changes in Adolescent Mental Health Before and During Different Phases of the COVID-19 Pandemic. *Stress Health* 2022;:smi.3179. doi:10/gqn2rd

42 Runge RA, Glaesmer H, Schmitz J, *et al.* Mental Health in Children of Immigrants in Germany: The Role of Socio-Demographic and Immigration-Related Characteristics. *J Child Fam Stud* 2022;**31**:155–68. doi:10/gqzdsm

43 Beiser M, Hou F, Hyman I, *et al.* Poverty, Family Process, and the Mental Health of Immigrant Children in Canada. *Am J Public Health* 2002;**92**:220–7. doi:10/cpfc8w

44 Kröger H, Pakpahan E, Hoffmann R. What Causes Health Inequality? A Systematic Review on the Relative Importance of Social Causation and Health Selection. *Eur J Public Health* 2015;**25**:951–60. doi:10/f75vg2

45 Hermansen AS. Moving Up or Falling Behind? Intergenerational Socioeconomic Transmission among Children of Immigrants in Norway. *Eur Sociol Rev* 2016;**32**:675–89. doi:10/gmjwfg

46 Elshahat S, Moffat T, Newbold KB. Understanding the Healthy Immigrant Effect in the Context of Mental Health Challenges: A Systematic Critical Review. *J Immigr Minor Health* 2022;**24**:1564–79. doi:10/gr9tcj

47 Tribble R, Kim P. Intergenerational Transmission of Poverty: How Low Socioeconomic Status Impacts the Neurobiology of Two Generations. In: Harrist AW, Gardner BC, eds. *Biobehavioral Markers in Risk and Resilience Research*. Cham: : Springer International Publishing 2019. 49–67. doi:10.1007/978-3-030-05952-1_4

48 Markussen S, Røed K. Economic Mobility Under Pressure. *J Eur Econ Assoc* 2020;**18**:1844–85.

For peer review only

doi:10/gqzkmt

49 Black SE, Devereux PJ, Salvanes KG. Why the apple doesn't fall far: Understanding intergenerational transmission of human capital. *Am Econ Rev* 2005;**95**:437–49. doi:10/fhd9j4

50 Dahl GB, Kostøl AR, Mogstad M. Family welfare cultures. *Q J Econ* 2014;**129**. doi:10/f6vfqh

51 Repetti RL, Taylor SE, Seeman TE. Risky families: family social environments and the mental and physical health of offspring. *Psychol Bull* 2002;**128**:330–66.

For peer review only

Acknowledgements

We would like to thank master's students Vilde Johanna Solheim Lie, Hedda Andrea Struksnæs Sørdal and Helene Angelica Ostojic whose theses in clinical psychology at the University of Bergen functioned as a first draft to this article. We would like to thank the participants and acknowledge the New Patterns project family-coordinators who conducted the mapping and supported the participants over time.

Declaration of interest

All authors declare that they have no conflict of interest.

Funding

The work was partly funded by a grant from the Research Council of Norway (number 295686).

For peer review only

Table 1.

Sociodemographic Characteristics of Participants at Baseline

	N	%
Highest education level in the family	152	
Not finished primary school	24	16 %
Primary school	54	36 %
Secondary school	52	34 %
Higher education	22	14 %
Family housing	152	
Owns residence	15	10 %
Rents residence	86	56 %
Rents public housing	51	34 %
Parental employment status	152	
Unemployed	119	78 %
Employed	33	22 %
Children's immigrant status	301	
Non-immigrant background	102	34 %
Immigrant background[a]	199	66 %
Child born in Norway	201	67%
Children's gender	301	
Female	162	54 %
Male	139	46 %
Children's Age (*M*, (*SD*))	10.76	(3.74)
Children's living arrangement	301	
Mother alone	113	38 %
Father alone	13	4 %
Mother and father	115	38 %
Lives with mother, visit father	50	17 %
Other (Shared residence, or lives with father and visit mother)	10	3 %

Note. [a]Immigrant or born in Norway with one or two immigrant parents.

For peer review only

Table 2.

Parent reported Strengths and Difficulties Score, Stratified by Sex and Migration background

	Total	Sex			Background		
	(1) Total (n = 153) Mean (SD)	(2) Girls (n = 61) Mean (SD)	(3) Boys (n = 92) Mean (SD)	(4) Mean difference Mean [95 % CI]	(5) Immigrant (n = 87) Mean (SD)	(6) Non-immigrant (n = 66) Mean (SD)	(7) Mean difference Mean [95 % CI]
Total difficulties	10.74	9.84	11.34	1.50	9.51	12.36	2.86**
	(6.68)	(6.79)	(6.58)	[-0.67, 3.67]	(6.26)	(6.93)	[0.73, 4.98]
Emotional symptoms	2.56	2.62	2.52	-0.10	2.18	3.06	0.88*
	(2.36)	(2.58)	(2.23)	[-0.89, 0.69]	(2.19)	(2.51)	[0.12, 1.64]
Conduct problems	2.00	1.79	2.14	0.35	1.53	2.62	1.09***
	(1.96)	(1.98)	(1.94)	[-0.28, 0.99]	(1.63)	(2.18)	[0.46, 1.72]
Hyperactivity	4.48	3.90	4.87	0.97*	4.08	5.02	0.93*
	(2.82)	(2.78)	(2.79)	[0.07, 1.87]	(2.65)	(2.97)	[0.03, 1.84]
Peer problems	1.69	1.52	1.80	0.28	1.71	1.67	-0.05
	(1.84)	(1.64)	(1.97)	[-0.30, 0.86]	(1.91)	(1.77)	[-0.63, 0.54]
Prosocial	8.69	9.18	8.37	-0.81**	8.99	8.30	-0.69*
	(1.78)	(1.31)	(1.97)	[-1.33, -0.29]	(1.56)	(1.98)	[-1.26, -0.11]
Impact	1.27	0.85	1.55	0.70	1.03	1.59	0.56
	(2.39)	(1.71)	(2.72)	[-0.00, 1.40]	(2.61)	(2.05)	[-0.18, 1.29]

Notes: Column 1: Mean score parent reported SDQ. Standard deviation (SD) in parentheses. Column 2: Mean score girls. SD in parentheses. Column 3: Mean score for boys. SD in parentheses. Column 4: Mean difference in SDQ-scores between boys and girls with 95% confidence intervals in brackets using t-test assuming unequal variance. Column 5: Mean score for children with immigrant background (immigrant or born in Norway with one or two immigrant parents). SD in parentheses. Column 6: Mean score for children with non-immigrant background (born in Norway with parents without immigrant background). SD in parentheses. Column 7: Mean difference in SDQ-scores between children with and without immigrant background with 95% confidence intervals in brackets using t-test assuming unequal variance.

* $p < 0.05$, ** $p < 0.01$, *** $p < 0.001$

For peer review only - http://bmjopen.bmj.com/site/about/guidelines.xhtml

Table 3.

Self-reported Strengths and Difficulties Score, Stratified by Sex and Migration background

	Total	Gender			Background		
	(1) Total (n = 148) Mean (SD)	(2) Girls (n = 78) Mean (SD)	(3) Boys (n = 70) Mean (SD)	(4) Mean difference Mean [95 % CI]	(5) Immigrant (n = 112) Mean (SD)	(6) Non-immigrant (n = 36) Mean (SD)	(7) Mean difference Mean [95 % CI]
Total difficulties	10.08	9.91	10.27	0.36	8.79	14.11	5.33***
	(5.57)	(5.60)	(5.56)	[-1.44, 2.16]	(5.14)	(4.91)	[3.46, 7.19]
Emotional symptoms	2.68	2.99	2.33	-0.66	2.17	4.25	2.08***
	(2.22)	(2.40)	(1.96)	[-1.36, 0.04]	(1.95)	(2.29)	[1.25, 2.91]
Conduct problems	1.52	1.50	1.54	0.04	1.35	2.06	0.71*
	(1.55)	(1.65)	(1.45)	[-0.46, 0.54]	(1.49)	(1.66)	[0.10, 1.31]
Hyperactivity	3.93	3.60	4.30	0.70	3.53	5.19	1.67***
	(2.47)	(2.46)	(2.45)	[-0.10, 1.49]	(2.42)	(2.24)	[0.81, 2.53]
Peer problems	1.95	1.82	2.10	0.28	1.74	2.61	0.87**
	(1.58)	(1.54)	(1.62)	[-0.23, 0.79]	(1.51)	(1.63)	[0.27, 1.47]
Prosocial	8.51	8.87	8.10	-0.77**	8.71	7.89	-0.82*
	(1.66)	(1.31)	(1.90)	[-1.30, -0.24]	(1.59)	(1.75)	[-1.46, -0.17]
Impact	0.97	1.13	0.78	-0.35	0.70	1.78	1.08*
	(2.08)	(2.16)	(1.99)	[-1.02,0.33]	(1.76)	(2.73)	[0.13,2.02]

Notes. Column 1: Mean score Self-reported SDQ. Standard deviation (SD) in parentheses. Column 2: Mean score girls. SD in parentheses. Column 3: Mean score for boys. SD in parentheses. Column 4: Mean difference in SDQ-scores between boys and girls with 95% confidence intervals in brackets using t-test assuming unequal variance. Column 5: Mean score for children with immigrant background (immigrant or born in Norway with one or two immigrant parents). SD in parentheses. Column 6: Mean score for children with non-immigrant background (born in Norway with parents without immigrant background). SD in parentheses. Column 7: Mean difference in SDQ-scores between children with and without immigrant background with 95% confidence intervals in brackets using t-test assuming unequal variance.

* $p < 0.05$, ** $p < 0.01$, *** $p < 0.001$

For peer review only

Table 4.

Results from Adjusted Multiple Linear Regression Analyses Predicting Scores on the Strengths and Difficulties Questionnaire

	(1) Total difficulties b (se)	(2) Emotional symptoms b (se)	(3) Conduct problems b (se)	(4) Hyperactivity b (se)	(5) Peer problems b (se)	(6) Prosocial b (se)	(7) Impact b (se)
Panel A: Parent reported SDQ							
Girl	-1.130	0.216	-0.191	-0.828	-0.327	0.704*	-0.666
	(1.253)	(0.422)	(0.344)	(0.514)	(0.311)	(0.280)	(0.386)
Age (mean centred)	0.369	0.200**	0.0752	-0.0389	0.133*	0.0336	0.190*
	(0.186)	(0.0622)	(0.0572)	(0.0698)	(0.0595)	(0.0502)	(0.0833)
Immigrant background	-2.805*	-1.014*	-1.083**	-0.747	0.0390	0.558	-0.539
	(1.254)	(0.431)	(0.369)	(0.500)	(0.329)	(0.323)	(0.411)
Time	-0.004	0.000	-0.001	-0.003	0.000	-0.001	0.001
	(0.007)	(0.003)	(0.002)	(0.003)	(0.002)	(0.002)	(0.002)
Constant	14.20***	3.520***	3.042***	5.464***	2.171***	8.259***	2.302***
	(1.275)	(0.432)	(0.411)	(0.485)	(0.423)	(0.323)	(0.514)
Observations	153	153	153	153	153	153	153
Panel B: Self-reported SDQ							
Girl	-0.586	0.550	-0.0630	-0.753	-0.320	0.808**	0.268
	(0.870)	(0.317)	(0.236)	(0.407)	(0.244)	(0.253)	(0.344)
Age (mean centred)	0.298	0.161*	-0.00598	0.0569	0.0863	-0.0497	0.122
	(0.189)	(0.0734)	(0.0534)	(0.0964)	(0.0681)	(0.0617)	(0.0899)
Immigrant background	-5.214***	-1.974***	-0.705**	-1.681***	-0.855**	0.850*	-1.017*
	(0.880)	(0.385)	(0.256)	(0.421)	(0.304)	(0.324)	(0.503)
Time	-0.008	-0.001	-0.002	-0.004	-0.001	0.001	0.002
	(0.006)	(0.003)	(0.002)	(0.003)	(0.002)	(0.002)	(0.003)
Constant	14.31***	3.504***	2.298***	5.862***	2.642***	7.436***	1.066
	(1.201)	(0.491)	(0.282)	(0.601)	(0.412)	(0.445)	(0.627)
Observations	148	148	148	148	148	148	147

Notes. Immigrant background is a dummy variable indicating if the child grows up in an immigrant household. *Age* is a continuous variable measuring age in years at the time when SDQ is reported, this variable was mean centred in the analyses. *Girl* is a dummy variable indication if the child is a girl. *Time* is a continuous variable measuring the number of days since a family coordinator was assigned to the family. Robust standard errors were clustered on the family level. The first line in each row presents the estimated coefficient, the second presents the standard errors (in parentheses).

* $p < 0.05$, ** $p < 0.01$, *** $p < 0.001$

For peer review only

Figure 1.

Self- and parent reported scores on the Strengths and Difficulties Questionnaire for immigrant and non-immigrant participants as deviations from the UK 4-band solution

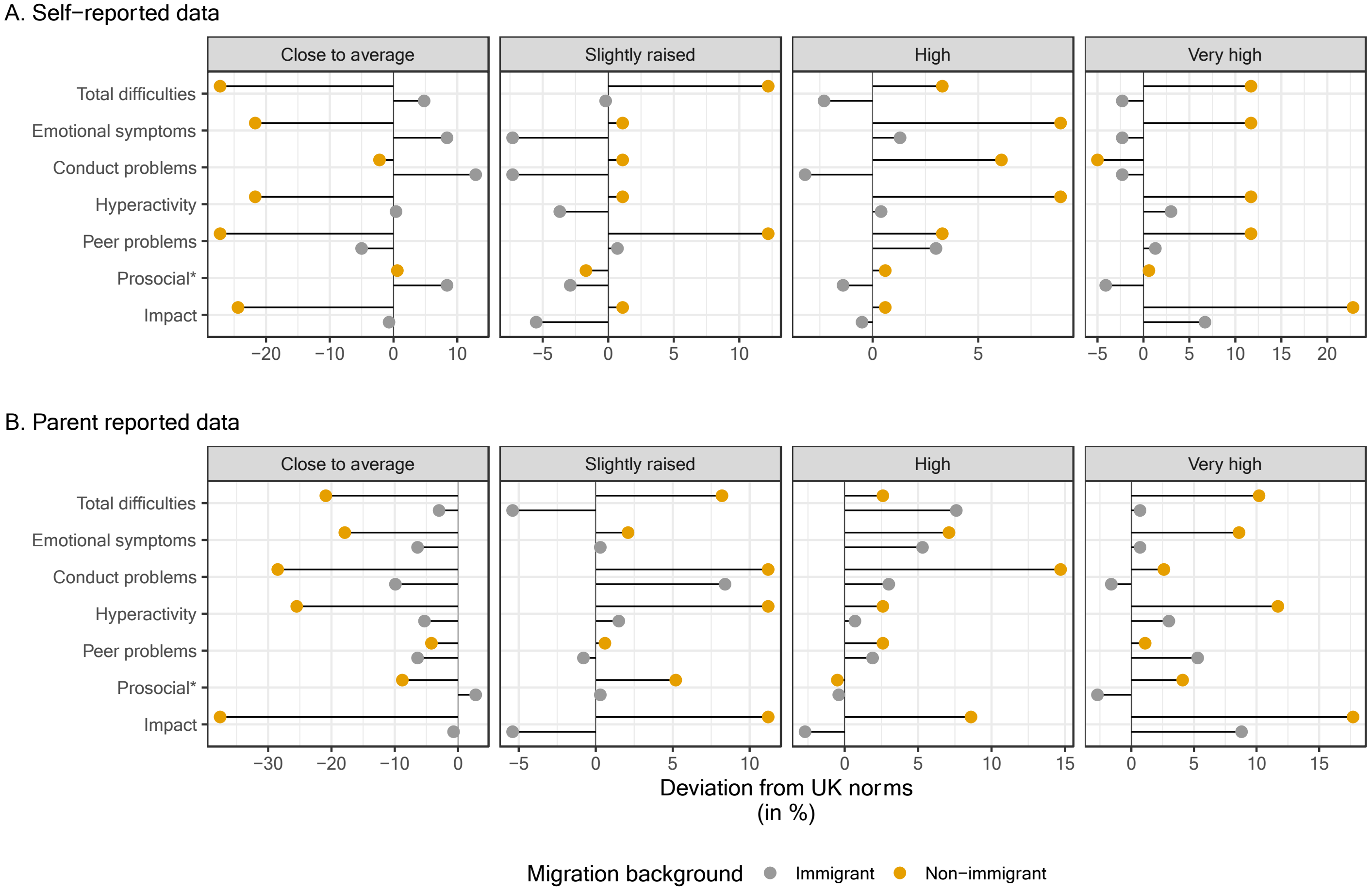

Note. The figure illustrates the scores in the current sample as deviations from the expected distribution based on the UK 4-band solution [26]. Category labels are printed on top of each facet in the plot. Negative values suggest that there are fewer than expected that score within a category based on UK norms, positive values indicate that more participants than expected score within that category whereas values of 0 suggest that scores correspond to distributions from the UK 4-band solution. Note that the scales of the x-axes are free.

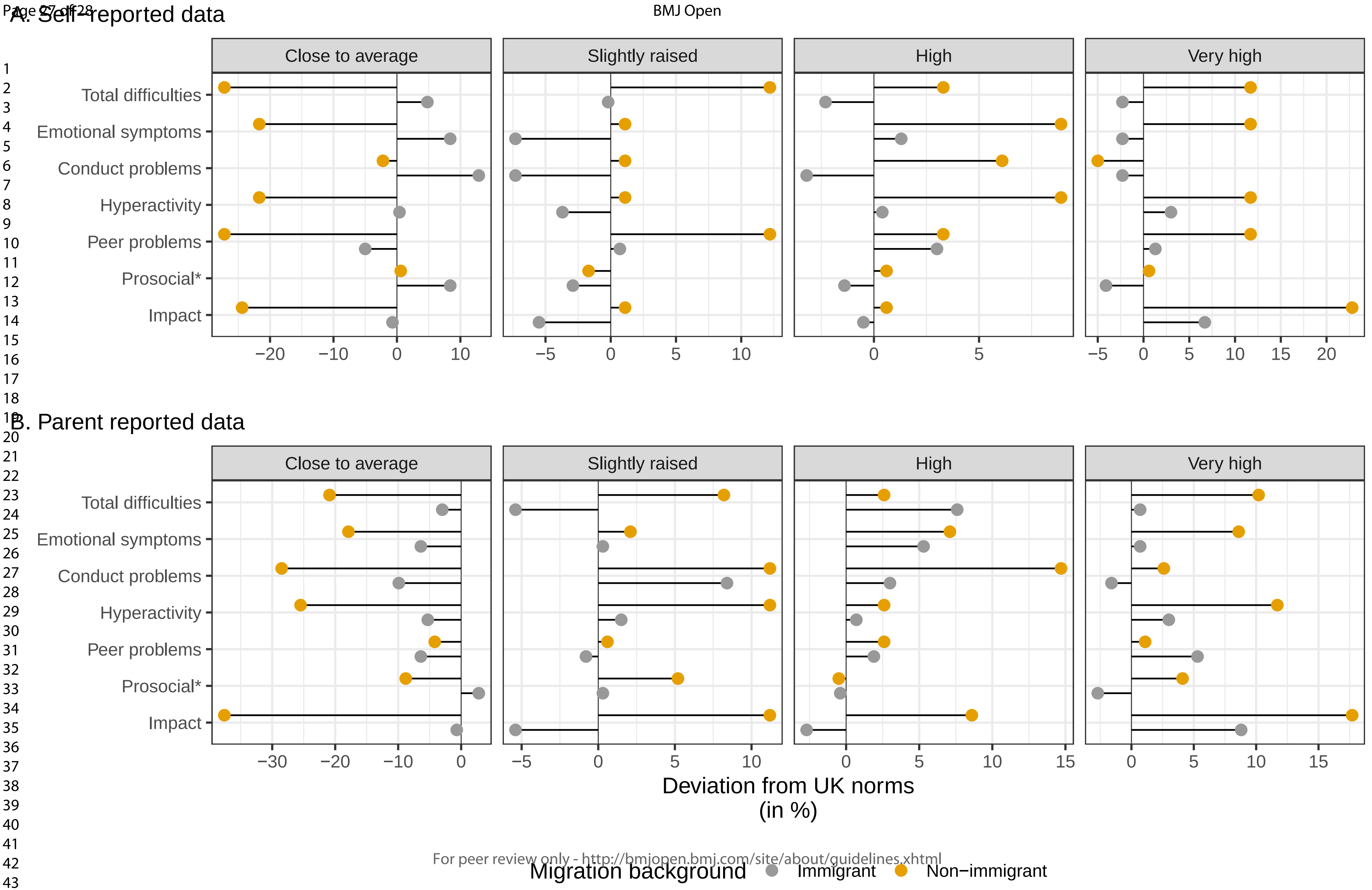
A. Self−reported data
Close to average
Slightly raised
High
Very high
Total difficulties
Emotional symptoms
Conduct problems
Hyperactivity
Peer problems
Prosocial*
Impact
−20
−10
0
10
−5
0
5
10
0
5
−5
0
5
10
15
20
B. Parent reported data
Close to average
Slightly raised
High
Very high
Total difficulties
Emotional symptoms
Conduct problems
Hyperactivity
Peer problems
Prosocial*
Impact
−30
−20
−10
0
−5
0
5
10
0
5
10
15
0
5
10
15
Deviation from UK norms
(in %)
Migration background
Immigrant
Non−immigrant

STROBE Statement—Checklist of items that should be included in reports of ***cross-sectional studies***

	Item No	Recommendation	Page No
**Title and abstract**	1	(*a*) Indicate the study's design with a commonly used term in the title or the abstract	2
		(*b*) Provide in the abstract an informative and balanced summary of what was done and what was found	2
**Introduction**			
Background/rationale	2	Explain the scientific background and rationale for the investigation being reported	3-4
Objectives	3	State specific objectives, including any prespecified hypotheses	4
**Methods**			
Study design	4	Present key elements of study design early in the paper	4
Setting	5	Describe the setting, locations, and relevant dates, including periods of recruitment, exposure, follow-up, and data collection	4
Participants	6	(*a*) Give the eligibility criteria, and the sources and methods of selection of participants	4
Variables	7	Clearly define all outcomes, exposures, predictors, potential confounders, and effect modifiers. Give diagnostic criteria, if applicable	5-6
Data sources/ measurement	8*	For each variable of interest, give sources of data and details of methods of assessment (measurement). Describe comparability of assessment methods if there is more than one group	5-6
Bias	9	Describe any efforts to address potential sources of bias	12
Study size	10	Explain how the study size was arrived at	4-5
Quantitative variables	11	Explain how quantitative variables were handled in the analyses. If applicable, describe which groupings were chosen and why	6
Statistical methods	12	(*a*) Describe all statistical methods, including those used to control for confounding	6
		(*b*) Describe any methods used to examine subgroups and interactions	6
		(*c*) Explain how missing data were addressed	6
		(*d*) If applicable, describe analytical methods taking account of sampling strategy	6
		(*e*) Describe any sensitivity analyses	6,11
**Results**			
Participants	13*	(a) Report numbers of individuals at each stage of study—eg numbers potentially eligible, examined for eligibility, confirmed eligible, included in the study, completing follow-up, and analysed	18
		(b) Give reasons for non-participation at each stage	na
		(c) Consider use of a flow diagram	na
Descriptive data	14*	(a) Give characteristics of study participants (eg demographic, clinical, social) and information on exposures and potential confounders	18
		(b) Indicate number of participants with missing data for each variable of interest	na
Outcome data	15*	Report numbers of outcome events or summary measures	20-22

For peer review only

Main results	16	(*a*) Give unadjusted estimates and, if applicable, confounder-adjusted estimates and their precision (eg, 95% confidence interval). Make clear which confounders were adjusted for and why they were included	22
		(*b*) Report category boundaries when continuous variables were categorized	na
		(*c*) If relevant, consider translating estimates of relative risk into absolute risk for a meaningful time period	na
Other analyses	17	Report other analyses done—eg analyses of subgroups and interactions, and sensitivity analyses	10
**Discussion**			
Key results	18	Summarise key results with reference to study objectives	9-10
Limitations	19	Discuss limitations of the study, taking into account sources of potential bias or imprecision. Discuss both direction and magnitude of any potential bias	11
Interpretation	20	Give a cautious overall interpretation of results considering objectives, limitations, multiplicity of analyses, results from similar studies, and other relevant evidence	9-13
Generalisability	21	Discuss the generalisability (external validity) of the study results	12
**Other information**			
Funding	22	Give the source of funding and the role of the funders for the present study and, if applicable, for the original study on which the present article is based	17

*Give information separately for exposed and unexposed groups.

Note: An Explanation and Elaboration article discusses each checklist item and gives methodological background and published examples of transparent reporting. The STROBE checklist is best used in conjunction with this article (freely available on the Web sites of PLoS Medicine at http://www.plosmedicine.org/, Annals of Internal Medicine at http://www.annals.org/, and Epidemiology at http://www.epidem.com/). Information on the STROBE Initiative is available at www.strobe-statement.org.

For peer review only

For peer review only - http://bmjopen.bmj.com/site/about/guidelines.xhtml

BMJ Open

Self- and parent-reported mental health in children from low-income families in Agder, Norway: Results from baseline measurements of New Patterns project participants

Journal:	*BMJ Open*
Manuscript ID	bmjopen-2023-076400.R1
Article Type:	Original research
Date Submitted by the Author:	24-Oct-2023
Complete List of Authors:	Bøe, Tormod; University of Bergen, Department of Psychosocial Science; NORCE Norwegian Research Centre AS Forskningsområde Helse, Regional Centre for Child and Youth Mental Health and Child Welfare<br>Ostojic, Helene Angelica; Vestre Viken HF, Drammen District Psychiatric Center, Mental Health and Substance Abuse; University of Bergen, Department of Psychosocial Science<br>Haraldstad, Kristin; University of Agder, Dep of Health and Nursing Sciences, Faculty of health and Sport Sciences, University of Agder, Norway<br>Abildsnes, Eirik; University of Agder, Dep of Psychosocial Health<br>Wilson, Philip; University of Aberdeen, Centre for Rural Health<br>Vigsnes, Kristine; Kristiansand Kommune<br>Mølland, Eirin; University of Agder, School of Business and Law; NORCE Norwegian Research Centre AS Forskningsomrade Samfunn
**Primary Subject Heading**:	Mental health
Secondary Subject Heading:	Epidemiology, Public health
Keywords:	SOCIAL MEDICINE, Health Equity, MENTAL HEALTH

SCHOLARONE™
Manuscripts

I, the Submitting Author has the right to grant and does grant on behalf of all authors of the Work (as defined in the below author licence), an exclusive licence and/or a non-exclusive licence for contributions from authors who are: i) UK Crown employees; ii) where BMJ has agreed a CC-BY licence shall apply, and/or iii) in accordance with the terms applicable for US Federal Government officers or employees acting as part of their official duties; on a worldwide, perpetual, irrevocable, royalty-free basis to BMJ Publishing Group Ltd ("BMJ") its licensees and where the relevant Journal is co-owned by BMJ to the co-owners of the Journal, to publish the Work in this journal and any other BMJ products and to exploit all rights, as set out in our licence.

The Submitting Author accepts and understands that any supply made under these terms is made by BMJ to the Submitting Author unless you are acting as an employee on behalf of your employer or a postgraduate student of an affiliated institution which is paying any applicable article publishing charge ("APC") for Open Access articles. Where the Submitting Author wishes to make the Work available on an Open Access basis (and intends to pay the relevant APC), the terms of reuse of such Open Access shall be governed by a Creative Commons licence – details of these licences and which Creative Commons licence will apply to this Work are set out in our licence referred to above.

Other than as permitted in any relevant BMJ Author's Self Archiving Policies, I confirm this Work has not been accepted for publication elsewhere, is not being considered for publication elsewhere and does not duplicate material already published. I confirm all authors consent to publication of this Work and authorise the granting of this licence.

For peer review only

Self- and parent-reported mental health in children from low-income families in Agder, Norway: Results from baseline measurements of New Patterns project participants

Tormod Bøe[a,b] *Helene Angelica Ostojic[a,c], Kristin Haraldstad[d], Eirik Abildsnes[e], Philip Wilson[f], Kristine Vigsnes[g] and Eirin Mølland[h,i]

[a] Department of Psychosocial Science, Faculty of Psychology, University of Bergen, Bergen, Norway,

[b] Regional Centre for Child and Youth Mental Health and Child Welfare, NORCE Norwegian Research Centre, Bergen, Norway

[c] Drammen District Psychiatric Center, Mental Health and Substance Abuse, Vestre Viken HC, Drammen, Norway

[d] Dep of Health and Nursing Sciences, Faculty of health and Sport Sciences, University of Agder, Norway

[e] Dep of Psychosocial Health, Faculty of Health and Sport Sciences, University of Agder, Norway

[f] Centre for Rural Health, Institute of Applied Health Sciences, University of Aberdeen, Scotland

[g] Kristiansand Municipality, Kristiansand, Norway

[h] School of Business and Law, University of Agder, Kristiansand, Norway

[i] NORCE Norwegian Research Centre, Bergen, Norway

*Corresponding author: Tormod Bøe, tormod.boe@uib.no

For peer review only

Strengths and limitations of this study

- This study presents baseline data on mental health problems gathered using a validated instrument among participants in a poverty intervention study conducted in southern Norway
- The study provides valuable information about a hard-to-reach sample, and includes participants with and without immigrant backgrounds
- Due to recruitment procedures, there is no control- or comparison group included in the survey
- The recruitment procedure may have biased the sample towards poor families with many difficulties, making the findings somewhat less generalisable

For peer review only

Abstract

Background. Poverty may pose risks to child and adolescent mental health, but few studies have reported on this association among children and adolescents in low-income families in Norway. **Methods.** Based on a sample participating in an intervention for low-income families in Norway, we report data from the survey administered at the start of the intervention. Mental health problems were measured using the Strengths and Difficulties Questionnaire (SDQ; self-report (SR) n = 148; parent/proxy-report (PR) n = 153, mean age = 10.8). Demographic and family characteristics were obtained from parent reported data. Results are presented by gender and migration background. Regression analysis was used to investigate the relative contribution of background factors to mental health symptoms. The distribution of scores is compared to UK norms.

Results. Participants reported relatively high scores on the SDQ Total Difficulties Scale (PR mean = 10.7; SR mean = 10.1). Participants with non-immigrant backgrounds scored considerably higher on the Total Difficulties Scale (PR mean difference = 2.9; SR 5.3) and on most other domains measured with the SDQ compared to their peers with immigration backgrounds. Participants generally scored higher than or equal to UK norms.

Conclusion. Participants in the current study had many symptoms of mental health problems, with large differences between those with and without a migrant background. Interventions for low-income families should be based on detailed knowledge about differences in family risks, resources and needs.

For peer review only

Introduction

Mental health problems in children and adolescents are common. Up to 20% of children and young adolescents have a mental disorder [1], and the prevalence may be rising, for girls in particular [2]. Mental health problems negatively impact young people's health and well-being, as well as their future educational prospects, employment and earnings [3]. Mental health problems also account for a substantial and increasing world-wide burden of ill-health [4].

Child and adolescent mental health problems are distributed unequally with regards to household income: More children and adolescents in families with low income have mental health problems compared to more affluent peers [5–7]. Children and adolescents with low socioeconomic status, (SES) are two to three times more likely to develop mental health problems than their peers with high SES [5,8]. Research suggests low income mainly influences children and adolescents indirectly, through constraining caregivers' abilities to deploy resources towards their development, and through increased family stress with detrimental consequences for parental mental health and parenting practices [9]. There may also be direct influences through toxic effects of poverty-related stress on the brains of children and adolescents, potentially influencing structure and function in brain areas involved in memory, emotion regulation, and executive function, with implications for mental health and cognitive functioning [10].

Migration background may also be related to elevated mental health problems in children and adolescents [11]. It was, for example, found that second-generation migrant children had higher rates of depression, anxiety and post-traumatic stress disorders compared to their native counterparts [12]. This is particularly relevant to the current study, as migrant families and their children make up a large portion of the low-income population in Norway [13].

The current study is based on data from *New Patterns*, an intervention study conducted in a sample of families with children younger than 17 purposefully recruited based on having household incomes below the low-income threshold in Norway (i.e. ≤ 60% of the equivalized population median income) as well as having longstanding needs for welfare services [14]. Families that are included in

For peer review only - http://bmjopen.bmj.com/site/about/guidelines.xhtml

New Patterns receive a family coordinator who is responsible for follow-up of all family members for a period of five years and coordinate services that are involved with the family. Norwegian register- and community-based observational studies have shown that children and adolescents from households with lower income and low socioeconomic status have a higher frequency of mental disorders relative to higher income peers [15]. Many children and adolescents with mental health problems nevertheless go undetected [16]. Furthermore, children from low-income families are less likely to participate in research and community programs compared to more affluent peers [17]. Therefore, register-based studies should be complemented by epidemiological studies directly targeting these low-income hard-to-reach samples to more fully capture the mental health status of children and adolescents who live in low-income families.

The aim of the study is to present results from the baseline mental health screening completed by participants in the New Patterns project. In presenting these data, we will provide results separately by informant; for younger children, their parent or a proxy completed the Strengths and Difficulties Questionnaire (SDQ), but older children completed it themselves. We also stratify our results by sex as differences in the trajectories of mental health problems among boys and girls are widely reported [18]. Finally, we present results separately for children and adolescents with and without immigrant background since previous studies have shown that ethnic minority children have higher mean SDQ scores compared to majority population samples in Norway [19].

Methods

Participants and procedures

The *New Patterns* project targets families with children aged 0-17 living in Norway with low household income and additional challenges requiring longstanding need for services [14]. Families were referred to the intervention from different service sectors within the municipalities (i.e., kindergarten, school, public health clinics, general practitioners, the Norwegian Labour and Welfare Administration (NAV), child protection services and mental health services). Each referral was subsequently discussed in a multidisciplinary intake-team who selected the families that were

For peer review only

considered eligible to participate based on their needs for coordinated longer-term follow-up. The study is conducted according to recommendations from the Norwegian Data Protection Services (record numbers 27435) and approved by the regional ethics committee of western Norway (approval number 249507).

Our sample consists of the baseline self-reported (number of participants (n) = 148, age mean (M) = 13.55, standard deviation (SD) = 1.95) and parent-reported (n = 153, age M = 8.07, SD = 3.01) SDQ questionnaires for children participating in the New Patterns project. The sample was restricted to reports that were gathered within 360 days after enrolment to avoid potentially measuring the effect of the intervention and for children in the age-range where Strengths and Difficulties Questionnaire SDQ is validated (4-17 years in the current study). The median time in the project when the SDQ was reported was 82 days. Twelve children were included in both the parent- and self-reported dat. As results are presented by informant, this has not resulted in any double counting or other violations of sound analytical nor statistical practices.

Patient and Public involvement

None

Instruments

Mental health problems

Mental health was assessed with parent- and self-report versions of the extended Norwegian translation of the Strengths and Difficulties Questionnaire (SDQ) [20]. The psychometric properties of the SDQ have been well-documented [21,22]. The Norwegian translations of the SDQ have shown strong psychometric properties for all informant versions (abstract in English available at the PSYKTESTBARN website: shorturl.at/DUVY2), and have also received support for administration to 18-19-year-olds [23]. The SDQ contains 25 items, each of which can be answered using: “certainly true”, “somewhat true”, or “not true”. They can be summed into five subscales each containing five items with a potential range of scores from 0 to 10: *conduct problems, hyperactivity/inattention,*

For peer review only

emotional problems, peer relationship problems, and *prosocial behaviour*. The first four subscales can be added together as a *Total Difficulties Scale*, with a potential range from 0 to 40. The extended SDQ includes an impact supplement with items measuring functional impairment regarding chronicity, distress, social impairment, and burden to others [20].

Sociodemographic variables

Background characteristics were measured when the family entered the New Patterns project. *Parental education* was assessed as the highest level of education completed by the mother or father. If at least one of the parents in the household was employed at the time of entering the project, we defined parental employment status to be *employed*. Children who were born in Norway with one or two parents who had immigrated to Norway or children who themselves had immigrated to Norway were defined as having an *immigrant background.* Children born in Norway whose parents did not have immigrant background were defined as having a *non-immigrant background.* We used information about year of birth and the date of answering the SDQ questionnaire to compute approximately the age at which the SDQ questionnaire was completed.

Statistical analyses

Mean differences with 95% confidence intervals (CI) in SDQ-scores between boys and girls, and between children with immigrant and non-immigrant background were analysed using *t*-tests assuming unequal variance. Self-reported and parent-reported data were analysed separately. We then proceeded to estimate the following regression model:

$$y_i = \beta_0 + \beta_1 girl_i + \beta_2 age_i + \beta_3 Immigrant_i + \beta_4 Time_i + \epsilon_i$$

Where y_i is the outcome variable; SDQ Total Difficulties Scale score and the five-subscales. The variable *girl* is a dummy variable indicating if the individual is a girl, *age* is a continuous variable measuring age in years at the time when SDQ was reported. This variable was mean centred in the analyses ($age_{centered}$ = age – mean(age)) to ease interpretability of the regression coefficients.

For peer review only - http://bmjopen.bmj.com/site/about/guidelines.xhtml

Immigrant is a dummy variable indicating if the child has immigrant background. *Time* is a continuous variable measuring the number of days since a family coordinator was assigned to the family. Robust standard errors were clustered on the family level. Stata version 17 was used for all analyses [24] whereas the R-package *ggplot2* [25] was used for making figures.

To compare the distribution of scores in our current sample with UK norms, we created cut-points corresponding to the UK 4-band solution [26]. These cut-points are based on percentile distributions in a large UK sample and use the categories: scores up to the 80th percentiles are labelled "Close to average", scores between 81st-90th percentiles are labelled "Slightly raised (/Slightly lowered)", 91st-95th percentile are labelled "High (/Low)" and scores higher than 95th percentile are labelled "Very high (/Very low)". The labels in parenthesis are used for the prosocial score which is reversed scored so that lower scores mean more problems.

Results

Representativeness

More participants from *New Patterns* had a at least one parent with immigrant background (66%) compared to the general low-income population (59.3%) [27]. A higher percentage of participants from *New Patterns* also lived in a single parent household, i.e., with mother alone or father alone (38.2%) compared to the general low-income population (36.1%). The parents' educational levels were comparable to the general low-income population in Norway [28].

Descriptive statistics

Most participants had parents with secondary education or lower (86%) as their highest education, lived in rented residences or public housing (90 %) and were unemployed (78%), see Table 1. The mean age of participating children and adolescents was 10.8 (SD = .7), most had an immigrant background (66%) and most lived in a household with either a mother and father (38%) or a single mother (38%).

[Table 1 near here]

For peer review only

Mental health problems by sex and immigrant background

Table 2 presents descriptive statistics and subgroup differences in SDQ scores reported by parents. The total sample mean was 10.7 (SD = 6.7), with the highest subscale problem score being hyperactivity (M = 4.5, SD = 2.8). Most children were rated high on prosocial skills by their parents with a mean close to 9. Boys were rated as having significantly more symptoms of hyperactivity than girls (mean difference .97 [95% CI = .07, 1.9]). Boys were also rated as significantly less prosocial than girls (mean difference -.81, [95% CI = -1.3, -.3]). Children from a non-migrant background were rated as having significantly higher Total Difficulties Scale scores (mean difference 2.86, [95% CI = .7, 5]). Non-immigrant children were also rated as having more symptoms of emotional difficulties, conduct problems, hyperactivity/inattention problems and were also rated as less prosocial than their peers with immigrant backgrounds. A sensitivity check of parent reported data revealed that there were minor differences between those with immigrant background who were born in Norway (*M* = 9.5, *SD* = 6.5) and those born outside of Norway (*M* = 9.5, *SD* = 5.8) on the Total SDQ difficulties score.

[Table 2 near here]

Self-reported SDQ scores are presented in Table 3. The total sample mean for SDQ total difficulties was about 10 (SD = 5.6), and the highest problem subscale was hyperactivity/inattention problems (M = 3.9, SD = 2.5). Boys rated themselves significantly less prosocial than girls (mean difference = -.8 [95% CI = -1.30, -0.24]. Non-immigrant children scored higher on all SDQ problem subscales (e.g., the mean difference on the Total Difficulties Scale was 5.3 [95% CI = 3.46, 7.19]) compared to their peers with immigrant background. Non-immigrant children also scored significantly lower on the prosocial scale (-.8 [95% CI = [-1.46, -0.17]) and they perceived their symptoms of mental health problems as significantly more impairing, as indicated by a higher impact score (mean difference = 1.1, [95% CI = 0.13, 2.02].

[Table 3 near here]

For peer review only

Predictors of mental health differences from multiple linear regression

The results from the multiple linear regression analysis on predictors of SDQ scores can be seen in Table 4.

[Table 4 near here]

In the adjusted regression analyses, immigrant background was a significant predictor of fewer total difficulties (*b* = -2.8, *se* = 1.3), symptoms of emotional problems (*b* = 1.01, *se* = 0.4) and conduct problems (*b* = -1.08, *se* = 0.4) in parent-reported data, and across all SDQ subscales in self-reported data (see Panel B in Table 4). Older age was associated with more emotional symptoms (*b* = 0.2, *se* = 0.1), peer problems (*b* = 0.04, *se* = 0.1) and higher impact scores (*b* = 0.19, *se* = 0.1) in parent-reported data, and with more emotional symptoms (*b* = 0.16, *se* = 0.1) in self-reported data. Being a girl was associated with more prosocial behaviour in both parent- (*b* = 0.7, *se* = 0.3) and self-reported (*b* = 0.8, *se* = 0.3) SDQ data.

Categorization of scores according to UK 4-band categories

To compare the distribution of scores in our current sample with UK norms, we created cut-points corresponding to the UK 4-band solution for self- and parent report for immigrant and non-immigrant participants (see Figure 1). Figure 1 illustrate the deviation in percentages from the UK norms for each of the 4-bands (*close to average*, *slightly raised*, *high* and *very high*) by informant.

[Figure 1 near here]

The self-reported SDQ scores (Panel A, Figure 1) show that fewer than expected non-immigrant children (yellow circles) score *close to average* whereas more score in the *slightly raised*, *high* and *very high* categories for most problems measured by the SDQ. For prosocial behaviours, the pattern of results closely resembles the UK-norms. A different pattern emerged for children with immigrant backgrounds (grey circles). More children with migrant background than expected score

For peer review only

near or in the *close to average range*, and fewer non-immigrant children obtain scores that are classified into the *slightly raised*, *high* and *very high* categories. Two exceptions were hyperactivity/inattention and impact, where more children than expected obtained scores in the *very high* category.

In parent reported data (Panel B, Figure 1) the patters of results were more similar for participants with and without immigrant background. Fewer children without immigrant background than expected were given scores in the close to average category, whereas more children obtained scores categorised as *slightly raised*, *high* or *very high*. The largest deviations from UK norms were obtained for non-immigrant participants (yellow circles) in the Very high category, with two-three times as many non-immigrant children as expected scoring into this category for Total difficulties, emotional symptoms, hyperactivity/inattention, peer problems and impact. For children with immigrant background, more than expected obtained very high scores on impact, peer problems and hyperactivity.

Discussion

The current study presents results from parent- and self-reported mental health baseline screening of child and adolescent participants from an intervention targeting low-income families in Norway. The results suggest that participants had many symptoms of mental health problems; older children had more symptoms of emotional and peer problems and higher impact scores as reported by their parents, and more self-reported emotional symptoms. A main finding in the current study was the existence of differences in mental health between participants with and without immigrant backgrounds, with native children having higher levels of problems across most domains.

When comparing the distribution of scores to the 4-band SDQ scoring solution based on UK norms, it appeared that non-immigrant participants obtained relatively higher scores, whereas immigrant participants obtained scores that were lower or more in alignment with the distribution of scores in the UK norm data. Importantly, previous studies have found lower SDQ scores among

For peer review only

Nordic children and adolescents compared to peers in other (European) countries [29], so the scores obtained in the current study are higher than what has been reported previously. The finding of more symptoms of mental health problems in this sample can probably be attributed to the risk associated with low income and the challenging living conditions that characterises this sample [14].

In general, participants obtained relatively high scores on parent reported total difficulties. In a previous review of the SDQ in Nordic countries, Obel et al. [30] report parent-reported SDQ scores in the range of 5.7-6.4 in 7- to 9-year-old children from the Nordic countries. More recently, Gunnardsdottir [31] found a mean parent reported total difficulties score of 7.7 (SD = 4.3) across a sample of more than 6000 Nordic 4-16-year-olds. Moreover, the average self-reported Total Difficulties Scale scores in the current study were also relatively high, and comparable to scores previously reported by Obel et al. for older Norwegian adolescents (13-15-year-olds) [30]. The pattern of findings related to age and gender was in line with previous findings using the SDQ [32] although the scores from the current study were higher. The results are in general also aligned with normative gender- and age-related changes in development of mental health problems. Participants with immigrant background scored lower than previously reported while non-immigrants scored higher [30], and non-immigrants scored relatively high on UK norms. The finding of lower scores among participants with immigrant backgrounds where somewhat surprising as earlier studies in Norway and elsewhere have suggested that migration background can be a risk factor for mental health problems [11–13,19]. In the broader literature, however, migration background has been associated with both lower and higher morbidity [33,34]. The relationship between migration background and mental health outcomes may vary in line with different factors, including individual characteristics, socioeconomic status, cultural adaptation, and the circumstances surrounding the migration process. Overall, it is crucial to recognize the diversity within immigrant populations. One explanation for higher levels of mental health problems may be that some immigrants face significant challenges before they migrate, such as war, violence, discrimination, or economic hardship in their home countries, potentially contributing to increased vulnerability to

For peer review only

mental ill-health [35]. Moreover, some migrants may have limited social support in their new country, which can contribute to feelings of isolation and loneliness, also potentially increasing the risk of mental health problems [36].

The low scores among migrant participants in the current study could be related to methodological challenges related to survey completion or to the functioning of the SDQ [37]. Other studies have found parent reported SDQ scores to be functionally equivalent across immigration status [38]. For the self-report version, one prior Norwegian study suggested that the total difficulties score worked well, but that there could be difficulties interpreting some of the subscale scores [39], but other studies have suggested that the subscales may work well among migrant participants [40]. Also, more than 50% of the migrant sample were born in Norway, suggesting they should be able to adequately complete the SDQ. A sensitivity check of parent reported data revealed that there were minor differences between those with immigrant background who were born in Norway and those born outside of Norway on the Total SDQ difficulties score.

Another explanation for the discrepancies from findings could be that prior studies of migration-related differences have failed to adequately account for socioeconomic- and other differences between participants with and without migration backgrounds [41]. Prior studies may therefore have misattributed SES-related differences in mental health problems to migration background [42].

The differences in mental health between the two samples could also be related to the levels of complexity surrounding the low-income situation for participants with and without migration background. Mental health problems in participants with migration backgrounds may mainly be linked to material deprivation related to the migration and resettling process [43]. Deprivation may arise because migrants may not be able to access work for various reasons (e.g., due to language difficulties, or their educational and/or professional qualifications not being acknowledged or recognized in their receiving society). Unemployment or underemployment may result in a household income too low to make ends meet for migrant families, causing stresses and strains on

For peer review only

families that may result in increased mental health symptoms [44]. Importantly however, these stresses may be more transient for some and expected to eventually alleviate, thereby protecting the families from dysfunction resulting in less severe mental health problems in children with immigrant backgrounds. Studies do for example document that children of migrants achieve higher educational attainment and earnings as adults, in comparison to their native peers with similar socioeconomic status [45]. It may also be that migrants on average have better health, including mental health, compared to the native population, in line with the "health migrant effect", although the evidence in support of this hypothesis appears to be inconclusive [46].

For participants without migration background, however, the low-income situation may be more intricate. Beiser et al. [43] have documented that children with non-immigrant backgrounds that live in low-income households have more mental health problems than their peers with immigrant backgrounds, and that these problems are mostly mediated by single-parent status, less effective parenting practices, parental depression and family dysfunction. This is in line with previous studies that document how poverty may track indirectly over generations through poor quality parenting related to economic stress, parental mental health problems and family conflicts [47], in addition to more direct effects through transmission of earnings levels, welfare culture and human capital [48–50]. Non-immigrant participants in poor families may therefore both inherit more risky family environments and genetic vulnerabilities [51] and grow up with in more generally impoverished surroundings accumulating their risks for developing mental health problems.

Strengths and limitations

Among the strengths of the current study is the recruitment of a relatively large hard-to-reach sample of low-income families in Norway that includes both participants with and without migration background. Another strength is the use of a validated instrument to measure mental health problems. The findings from the current study should also been interpreted considering certain limitations; some of which regards the representativeness of the sample. Due to the recruitment

For peer review only - http://bmjopen.bmj.com/site/about/guidelines.xhtml

procedure, the sample of participants may have more difficulties compared to a regular low-income sample of Norwegian families, and the results may therefore not readily generalize to this population. Finally, although the sample was large compared to other studies of low-income families in Norway, a larger sample would have resulted in more precise point estimates from the statistical comparisons.

Conclusion

Child and adolescent participants participating in an intervention targeting low-income families in Norway had many symptoms of mental health problems at baseline, and participants with non-immigrant background had more severe problems. There is a need for more detailed assessments of the characteristics of families where children have significant mental health problems to better understand the mechanisms underlying the development of mental health problems in children who grow up in low-income families. This in turn could allow development of interventions that are better tailored to the different needs of the families and the children that grow up in these families.

Contributorship statement
TB, HAO, KH, EA, PW, KV and EM have contributed by making substantial contributions to the conception or design of the work and the acquisition of data for the work. TB, HAO and EM drafted the work and analysed the data, TB, HAO, KH, EA, PW, KV and EM contributed to interpretation of the work, revised it critically for important intellectual content, and gave final approval of the version to be published.

Competing interests
All authors declare that they have no conflict of interest.

Funding
The work was partly funded by a grant from the Research Council of Norway (number 295686).

Data sharing statement
There are legal and ethical restrictions on sharing the dataset used for the present manuscript. Access to the dataset require application and approval by Regional Committee for Medical and Health Research Ethics (REC) in Western Norway and the Norwegian Centre for Research Data (NSD). In addition, Norwegian Health research legislation and the Norwegian Ethics committees require explicit consent from participants to transfer health research data outside of Norway. In this specific case, ethics approval is also contingent on storing the research data on secure storage facilities located in our research institution.

For peer review only

Acknowledgements

We would like to thank master's students Vilde Johanna Solheim Lie, Hedda Andrea Struksnæs Sørdal and Helene Angelica Ostojic whose theses in clinical psychology at the University of Bergen functioned as a first draft to this article. We would like to thank the participants and acknowledge the New Patterns project family-coordinators who conducted the mapping and supported the participants over time.

For peer review only

References

1 Polanczyk GV, Salum GA, Sugaya LS, *et al.* Annual research review: a meta-analysis of the worldwide prevalence of mental disorders in children and adolescents. *J Child Psychol Psychiatry Allied Discip* 2015;**56**:345–65. doi:10/gdsndp

2 Collishaw S. Annual Research Review: Secular trends in child and adolescent mental health. *J Child Psychol Psychiatry* 2015;**56**:370–93. doi:10/f68wxn

3 Evensen M, Lyngstad TH, Melkevik O, *et al.* Adolescent mental health and earnings inequalities in adulthood: evidence from the Young-HUNT Study. *J Epidemiol Community Health* 2017;**71**:201–6. doi:10/f9kpvg

4 Murray CJL, Vos T, Lozano R, *et al.* Disability-adjusted life years (DALYs) for 291 diseases and injuries in 21 regions, 1990–2010: a systematic analysis for the Global Burden of Disease Study 2010. *The Lancet* 2012;**380**:2197–223. doi:10/f2f8rw

5 Reiss F. Socioeconomic inequalities and mental health problems in children and adolescents: a systematic review. *Soc Sci Med* 2013;**90**:24–31. doi:10/f43jnn

6 Barry SJE, Marryat L, Thompson L, *et al.* Mapping area variability in social and behavioural difficulties among Glasgow pre-schoolers: linkage of a survey of pre-school staff with routine monitoring data: Mapping area variability. *Child Care Health Dev* 2015;**41**:853–64. doi:10/f77dr6

7 Reiss F, Meyrose A-K, Otto C, *et al.* Socioeconomic status, stressful life situations and mental health problems in children and adolescents: Results of the German BELLA cohort-study. *PLOS ONE* 2019;**14**:e0213700. doi:10/gfz4x8

8 Marryat L, Thompson L, Minnis H, *et al.* Primary schools and the amplification of social differences in child mental health: a population-based cohort study. *J Epidemiol Community Health* 2018;**72**:27–33. doi:10/ggk725

9 Gershoff ET, Aber JL, Raver CC, *et al.* Income is not enough: incorporating material hardship into models of income associations with parenting and child development. *Child Dev* 2007;**78**:70–95. doi:10/gw5

10 Johnson SB, Riis JL, Noble KG. State of the Art Review: Poverty and the Developing Brain. *Pediatrics* 2016;:peds.2015-3075. doi:10/f8gmmh

11 Belhadj Kouider E, Koglin U, Petermann F. Emotional and behavioral problems in migrant children and adolescents in Europe: a systematic review. *Eur Child Adolesc Psychiatry* 2014;**23**:373–91. doi:10/gmbft5

12 Ceri V, Özlü-Erkilic Z, Özer Ü, *et al.* Mental health problems of second generation children and adolescents with migration background. *Int J Psychiatry Clin Pract* 2017;**21**:142–7. doi:10/gr8sp3

13 Povlsen L, Regber S, Fosse E, *et al.* Economic poverty among children and adolescents in the Nordic countries. *Scand J Public Health* 2018;**46**:30–7. doi:10/gc8vg2

14 Mølland E, Vigsnes KL, Bøe T, *et al.* The New Patterns study: coordinated measures to combat child poverty. *Scand J Public Health* 2020;:1403494820956452. doi:10/ghbm4k

15 Kinge JM, Øverland S, Flatø M, *et al.* Parental income and mental disorders in children and adolescents: prospective register-based study. *Int J Epidemiol* Published Online First: 11 May 2021. doi:10/gj7skq

16 Heiervang E, Stormark KM, Lundervold AJ, *et al.* Psychiatric disorders in Norwegian 8- to 10-year-

For peer review only

olds. *J Am Acad Child Adolesc Psychiatry* 2007;**46**:438–47. doi:10/d8kr3v

17 Drummond J, Schnirer L, So S, *et al.* The protocol for the Families First Edmonton trial (FFE): a randomized community-based trial to compare four service integration approaches for families with low-income. *BMC Health Serv Res* 2014;**14**:223. doi:10/f56s6h

18 Patalay P, Fitzsimons E. Development and predictors of mental ill-health and wellbeing from childhood to adolescence. *Soc Psychiatry Psychiatr Epidemiol* 2018;**53**:1311–23. doi:10/gfkkz2

19 Sagatun Å, Lien L, Søgaard AJ, *et al.* Ethnic Norwegian and ethnic minority adolescents in Oslo, Norway: A longitudinal study comparing changes in mental health. *Soc Psychiatry Psychiatr Epidemiol* 2008;**43**:87–95. doi:10/c7g4dt

20 Goodman R. The extended version of the strengths and difficulties questionnaire as a guide to child. *J Child Psychol Psychiatry Allied Discip* 1999;**40**:791. doi:10/dk7tcs

21 Muris P, Meesters C, Van den Berg F. The strengths and difficulties questionnaire (SDQ) further evidence for its reliability and validity in a community sample of dutch children and adolescents. *Eur Child Adolesc Psychiatry* 2003;**12**:1–8. doi:10/fc65c3

22 Ortuño-sierra J, Fonseca-pedrero E, Aritio-solana R, *et al.* New evidence of factor structure and measurement invariance of the sdq across five european nations. *Eur Child Adolesc Psychiatry* 2015;**24**:1523–34. doi:10/f7z84r

23 Bøe T, Hysing M, Skogen JC, *et al.* The strengths and difficulties questionnaire (SDQ): factor structure and gender equivalence in Norwegian adolescents. *PLoS One* 2016;**11**:1–15. doi:10/f9v8vm

24 StataCorp. Stata Statistical Software. 2022.

25 Wickham H. *ggplot2: Elegant Graphics for Data Analysis*. Springer-Verlag New York 2016. https://ggplot2.tidyverse.org

26 Youthinmind.org. Scoring the Strengths & Difficulties Questionnaire for age 4-17 or 18+. Scoring SDQ. 2016.https://www.sdqinfo.org/py/sdqinfo/c0.py (accessed 9 Oct 2022).

27 Normann TM, Epland J. Fremdeles 115 000 barn med vedvarende lavinntekt i 2020. SSB. 2022.https://www.ssb.no/inntekt-og-forbruk/inntekt-og-formue/statistikk/inntekts-og-formuesstatistikk-for-husholdninger/artikler/fremdeles-115-000-barn-med-vedvarende-lavinntekt-i-2020 (accessed 20 Sep 2022).

28 Bufdir. Barnefattigdom. 2022.https://bufdir.no/Statistikk_og_analyse/Barnefattigdom/#/4204 (accessed 20 Sep 2022).

29 Elberling H, Linneberg A, Olsen EM, *et al.* The prevalence of sdq-measured mental health problems at age 5-7 years and identification of predictors from birth to preschool age in a danish birth cohort: the copenhagen child cohort 2000. *Eur Child Adolesc Psychiatry* 2010;**19**:725–35. doi:10/bczc5d

30 Obel C, Heiervang E, Rodriguez A, *et al.* The Strengths and Difficulties questionnaire in the Nordic countries. *Eur Child Adolesc Psychiatry Suppl* 2004;**13**:II32-9. doi:10/d4bhcq

31 Gunnarsdottir H, Hensing G, Povlsen L, *et al.* Relative deprivation in the Nordic countries-child mental health problems in relation to parental financial stress. *Eur J Public Health* 2016;**26**:277–82. doi:10/f3m7dz

32 Niclasen J, Teasdale TW, Andersen AMN, *et al.* Psychometric properties of the danish strength and difficulties questionnaire: the sdq assessed for more than 70,000 raters in four different cohorts. *PLoS One* 2012;**7**:e32025. doi:10/ggmjt3

For peer review only

33 Sam DL, Vedder P, Liebkind K, *et al.* Immigration, acculturation and the paradox of adaptation in Europe. *Eur J Dev Psychol* 2008;**5**:138–58. doi:10/c37r49

34 Schwartz SJ, Unger JB, Zamboanga BL, *et al.* Rethinking the concept of acculturation: Implications for theory and research. *Am Psychol* 2010;**65**:237–51. doi:10/c97xbh

35 Lebano A, Hamed S, Bradby H, *et al.* Migrants' and refugees' health status and healthcare in Europe: a scoping literature review. *BMC Public Health* 2020;**20**:1039. doi:10/gjfj68

36 Gleeson C, Frost R, Sherwood L, *et al.* Post-migration factors and mental health outcomes in asylum-seeking and refugee populations: a systematic review. *Eur J Psychotraumatology* 2020;**11**:1793567. doi:10/gm6dtn

37 Maldonado BN, Chandna J, Gladstone M. A systematic review of tools used to screen and assess for externalising behavior symptoms in low and middle income settings. *Glob Ment Health* 2019;**6**:Article e13. doi:10/gmwdx6

38 Runge RA, Soellner R. Measuring children's emotional and behavioural problems: are SDQ parent reports from native and immigrant parents comparable? *Child Adolesc Psychiatry Ment Health* 2019;**13**:1–15. doi:10/gqzdsj

39 Richter J, Sagatun $\backslash$AAse, Heyerdahl S, *et al.* The Strengths and Difficulties Questionnaire (SDQ) - Self-Report. an Analysis of Its Structure in a Multiethnic Urban Adolescent Sample. *J Child Psychol Psychiatry Allied Discip* 2011;**52**:1002–11. doi:10/d7wbhg

40 Stevanovic D, Urbán R, Atilola O, *et al.* Does the Strengths and Difficulties Questionnaire – self report yield invariant measurements across different nations? Data from the International Child Mental Health Study Group. *Epidemiol Psychiatr Sci* 2015;**24**:323–34. doi:10/gd59p2

41 Stevens GWJM, Buyukcan-Tetik A, Maes M, *et al.* Examining Socioeconomic Disparities in Changes in Adolescent Mental Health Before and During Different Phases of the COVID-19 Pandemic. *Stress Health* 2022;:smi.3179. doi:10/gqn2rd

42 Runge RA, Glaesmer H, Schmitz J, *et al.* Mental Health in Children of Immigrants in Germany: The Role of Socio-Demographic and Immigration-Related Characteristics. *J Child Fam Stud* 2022;**31**:155–68. doi:10/gqzdsm

43 Beiser M, Hou F, Hyman I, *et al.* Poverty, Family Process, and the Mental Health of Immigrant Children in Canada. *Am J Public Health* 2002;**92**:220–7. doi:10/cpfc8w

44 Kröger H, Pakpahan E, Hoffmann R. What Causes Health Inequality? A Systematic Review on the Relative Importance of Social Causation and Health Selection. *Eur J Public Health* 2015;**25**:951–60. doi:10/f75vg2

45 Hermansen AS. Moving Up or Falling Behind? Intergenerational Socioeconomic Transmission among Children of Immigrants in Norway. *Eur Sociol Rev* 2016;**32**:675–89. doi:10/gmjwfg

46 Elshahat S, Moffat T, Newbold KB. Understanding the Healthy Immigrant Effect in the Context of Mental Health Challenges: A Systematic Critical Review. *J Immigr Minor Health* 2022;**24**:1564–79. doi:10/gr9tcj

47 Tribble R, Kim P. Intergenerational Transmission of Poverty: How Low Socioeconomic Status Impacts the Neurobiology of Two Generations. In: Harrist AW, Gardner BC, eds. *Biobehavioral Markers in Risk and Resilience Research*. Cham: : Springer International Publishing 2019. 49–67. doi:10.1007/978-3-030-05952-1_4

48 Markussen S, Røed K. Economic Mobility Under Pressure. *J Eur Econ Assoc* 2020;**18**:1844–85.

For peer review only

doi:10/gqzkmt

49 Black SE, Devereux PJ, Salvanes KG. Why the apple doesn't fall far: Understanding intergenerational transmission of human capital. *Am Econ Rev* 2005;**95**:437–49. doi:10/fhd9j4

50 Dahl GB, Kostøl AR, Mogstad M. Family welfare cultures. *Q J Econ* 2014;**129**. doi:10/f6vfqh

51 Repetti RL, Taylor SE, Seeman TE. Risky families: family social environments and the mental and physical health of offspring. *Psychol Bull* 2002;**128**:330–66.

For peer review only

Table 1.

Sociodemographic Characteristics of Participants at Baseline

	N	%
Highest education level in the family	152	
Not finished primary school	24	16 %
Primary school	54	36 %
Secondary school	52	34 %
Higher education	22	14 %
Family housing	152	
Owns residence	15	10 %
Rents residence	86	56 %
Rents public housing	51	34 %
Parental employment status	152	
Unemployed	119	78 %
Employed	33	22 %
Children's immigrant status	301	
Non-immigrant background	102	34 %
Immigrant background[a]	199	66 %
Child born in Norway	201	67%
Children's gender	301	
Female	162	54 %
Male	139	46 %
Children's Age (*M*, (*SD*))	10.76	(3.74)
Children's living arrangement	301	
Mother alone	113	38 %
Father alone	13	4 %
Mother and father	115	38 %
Lives with mother, visit father	50	17 %
Other (Shared residence, or lives with father and visit mother)	10	3 %

Note. [a]Immigrant or born in Norway with one or two immigrant parents.

For peer review only

Table 2.

Parent reported Strengths and Difficulties Score, Stratified by Sex and Migration background

	Total	Sex			Background		
	(1) Total (n = 153) Mean (SD)	(2) Girls (n = 61) Mean (SD)	(3) Boys (n = 92) Mean (SD)	(4) Mean difference Mean [95 % CI]	(5) Immigrant (n = 87) Mean (SD)	(6) Non-immigrant (n = 66) Mean (SD)	(7) Mean difference Mean [95 % CI]
Total difficulties	10.74	9.84	11.34	1.50	9.51	12.36	2.86**
	(6.68)	(6.79)	(6.58)	[-0.67, 3.67]	(6.26)	(6.93)	[0.73, 4.98]
Emotional symptoms	2.56	2.62	2.52	-0.10	2.18	3.06	0.88*
	(2.36)	(2.58)	(2.23)	[-0.89, 0.69]	(2.19)	(2.51)	[0.12, 1.64]
Conduct problems	2.00	1.79	2.14	0.35	1.53	2.62	1.09***
	(1.96)	(1.98)	(1.94)	[-0.28, 0.99]	(1.63)	(2.18)	[0.46, 1.72]
Hyperactivity	4.48	3.90	4.87	0.97*	4.08	5.02	0.93*
	(2.82)	(2.78)	(2.79)	[0.07, 1.87]	(2.65)	(2.97)	[0.03, 1.84]
Peer problems	1.69	1.52	1.80	0.28	1.71	1.67	-0.05
	(1.84)	(1.64)	(1.97)	[-0.30, 0.86]	(1.91)	(1.77)	[-0.63, 0.54]
Prosocial	8.69	9.18	8.37	-0.81**	8.99	8.30	-0.69*
	(1.78)	(1.31)	(1.97)	[-1.33, -0.29]	(1.56)	(1.98)	[-1.26, -0.11]
Impact	1.27	0.85	1.55	0.70	1.03	1.59	0.56
	(2.39)	(1.71)	(2.72)	[-0.00, 1.40]	(2.61)	(2.05)	[-0.18, 1.29]

Notes: Column 1: Mean score parent reported SDQ. Standard deviation (SD) in parentheses. Column 2: Mean score girls. SD in parentheses. Column 3: Mean score for boys. SD in parentheses. Column 4: Mean difference in SDQ-scores between boys and girls with 95% confidence intervals in brackets using t-test assuming unequal variance. Column 5: Mean score for children with immigrant background (immigrant or born in Norway with one or two immigrant parents). SD in parentheses. Column 6: Mean score for children with non-immigrant background (born in Norway with parents without immigrant background). SD in parentheses. Column 7: Mean difference in SDQ-scores between children with and without immigrant background with 95% confidence intervals in brackets using t-test assuming unequal variance.

* $p < 0.05$, ** $p < 0.01$, *** $p < 0.001$

For peer review only - http://bmjopen.bmj.com/site/about/guidelines.xhtml

Table 3.

Self-reported Strengths and Difficulties Score, Stratified by Sex and Migration background

	Total	Gender			Background		
	(1) Total (n = 148) Mean (SD)	(2) Girls (n = 78) Mean (SD)	(3) Boys (n = 70) Mean (SD)	(4) Mean difference Mean [95 % CI]	(5) Immigrant (n = 112) Mean (SD)	(6) Non-immigrant (n = 36) Mean (SD)	(7) Mean difference Mean [95 % CI]
Total difficulties	10.08	9.91	10.27	0.36	8.79	14.11	5.33***
	(5.57)	(5.60)	(5.56)	[-1.44, 2.16]	(5.14)	(4.91)	[3.46, 7.19]
Emotional symptoms	2.68	2.99	2.33	-0.66	2.17	4.25	2.08***
	(2.22)	(2.40)	(1.96)	[-1.36, 0.04]	(1.95)	(2.29)	[1.25, 2.91]
Conduct problems	1.52	1.50	1.54	0.04	1.35	2.06	0.71*
	(1.55)	(1.65)	(1.45)	[-0.46, 0.54]	(1.49)	(1.66)	[0.10, 1.31]
Hyperactivity	3.93	3.60	4.30	0.70	3.53	5.19	1.67***
	(2.47)	(2.46)	(2.45)	[-0.10, 1.49]	(2.42)	(2.24)	[0.81, 2.53]
Peer problems	1.95	1.82	2.10	0.28	1.74	2.61	0.87**
	(1.58)	(1.54)	(1.62)	[-0.23, 0.79]	(1.51)	(1.63)	[0.27, 1.47]
Prosocial	8.51	8.87	8.10	-0.77**	8.71	7.89	-0.82*
	(1.66)	(1.31)	(1.90)	[-1.30, -0.24]	(1.59)	(1.75)	[-1.46, -0.17]
Impact	0.97	1.13	0.78	-0.35	0.70	1.78	1.08*
	(2.08)	(2.16)	(1.99)	[-1.02,0.33]	(1.76)	(2.73)	[0.13,2.02]

Notes. Column 1: Mean score Self-reported SDQ. Standard deviation (SD) in parentheses. Column 2: Mean score girls. SD in parentheses. Column 3: Mean score for boys. SD in parentheses. Column 4: Mean difference in SDQ-scores between boys and girls with 95% confidence intervals in brackets using t-test assuming unequal variance. Column 5: Mean score for children with immigrant background (immigrant or born in Norway with one or two immigrant parents). SD in parentheses. Column 6: Mean score for children with non-immigrant background (born in Norway with parents without immigrant background). SD in parentheses. Column 7: Mean difference in SDQ-scores between children with and without immigrant background with 95% confidence intervals in brackets using t-test assuming unequal variance.

* $p < 0.05$, ** $p < 0.01$, *** $p < 0.001$

For peer review only

Table 4.

Results from Adjusted Multiple Linear Regression Analyses Predicting Scores on the Strengths and Difficulties Questionnaire

	(1) Total difficulties b (se)	(2) Emotional symptoms b (se)	(3) Conduct problems b (se)	(4) Hyperactivity b (se)	(5) Peer problems b (se)	(6) Prosocial b (se)	(7) Impact b (se)
Panel A: Parent reported SDQ							
Girl	-1.130	0.216	-0.191	-0.828	-0.327	0.704*	-0.666
	(1.253)	(0.422)	(0.344)	(0.514)	(0.311)	(0.280)	(0.386)
Age (mean centred)	0.369	0.200**	0.0752	-0.0389	0.133*	0.0336	0.190*
	(0.186)	(0.0622)	(0.0572)	(0.0698)	(0.0595)	(0.0502)	(0.0833)
Immigrant background	-2.805*	-1.014*	-1.083**	-0.747	0.0390	0.558	-0.539
	(1.254)	(0.431)	(0.369)	(0.500)	(0.329)	(0.323)	(0.411)
Time	-0.004	0.000	-0.001	-0.003	0.000	-0.001	0.001
	(0.007)	(0.003)	(0.002)	(0.003)	(0.002)	(0.002)	(0.002)
Constant	14.20***	3.520***	3.042***	5.464***	2.171***	8.259***	2.302***
	(1.275)	(0.432)	(0.411)	(0.485)	(0.423)	(0.323)	(0.514)
Observations	153	153	153	153	153	153	153
Panel B: Self-reported SDQ							
Girl	-0.586	0.550	-0.0630	-0.753	-0.320	0.808**	0.268
	(0.870)	(0.317)	(0.236)	(0.407)	(0.244)	(0.253)	(0.344)
Age (mean centred)	0.298	0.161*	-0.00598	0.0569	0.0863	-0.0497	0.122
	(0.189)	(0.0734)	(0.0534)	(0.0964)	(0.0681)	(0.0617)	(0.0899)
Immigrant background	-5.214***	-1.974***	-0.705**	-1.681***	-0.855**	0.850*	-1.017*
	(0.880)	(0.385)	(0.256)	(0.421)	(0.304)	(0.324)	(0.503)
Time	-0.008	-0.001	-0.002	-0.004	-0.001	0.001	0.002
	(0.006)	(0.003)	(0.002)	(0.003)	(0.002)	(0.002)	(0.003)
Constant	14.31***	3.504***	2.298***	5.862***	2.642***	7.436***	1.066
	(1.201)	(0.491)	(0.282)	(0.601)	(0.412)	(0.445)	(0.627)
Observations	148	148	148	148	148	148	147

Notes. Immigrant background is a dummy variable indicating if the child grows up in an immigrant household. *Age* is a continuous variable measuring age in years at the time when SDQ is reported, this variable was mean centred in the analyses. *Girl* is a dummy variable indication if the child is a girl. *Time* is a continuous variable measuring the number of days since a family coordinator was assigned to the family. Robust standard errors were clustered on the family level. The first line in each row presents the estimated coefficient, the second presents the standard errors (in parentheses).

* $p < 0.05$, ** $p < 0.01$, *** $p < 0.001$

For peer review only

Figure 1.

Self- and parent reported scores on the Strengths and Difficulties Questionnaire for immigrant and non-immigrant participants as deviations from the UK 4-band solution

For peer review only

Note. The figure illustrates the scores in the current sample as deviations from the expected distribution based on the UK 4-band solution [26]. Category labels are printed on top of each facet in the plot. Negative values suggest that there are fewer than expected that score within a category based on UK norms, positive values indicate that more participants than expected score within that category whereas values of 0 suggest that scores correspond to distributions from the UK 4-band solution. Note that the scales of the x-axes are free.

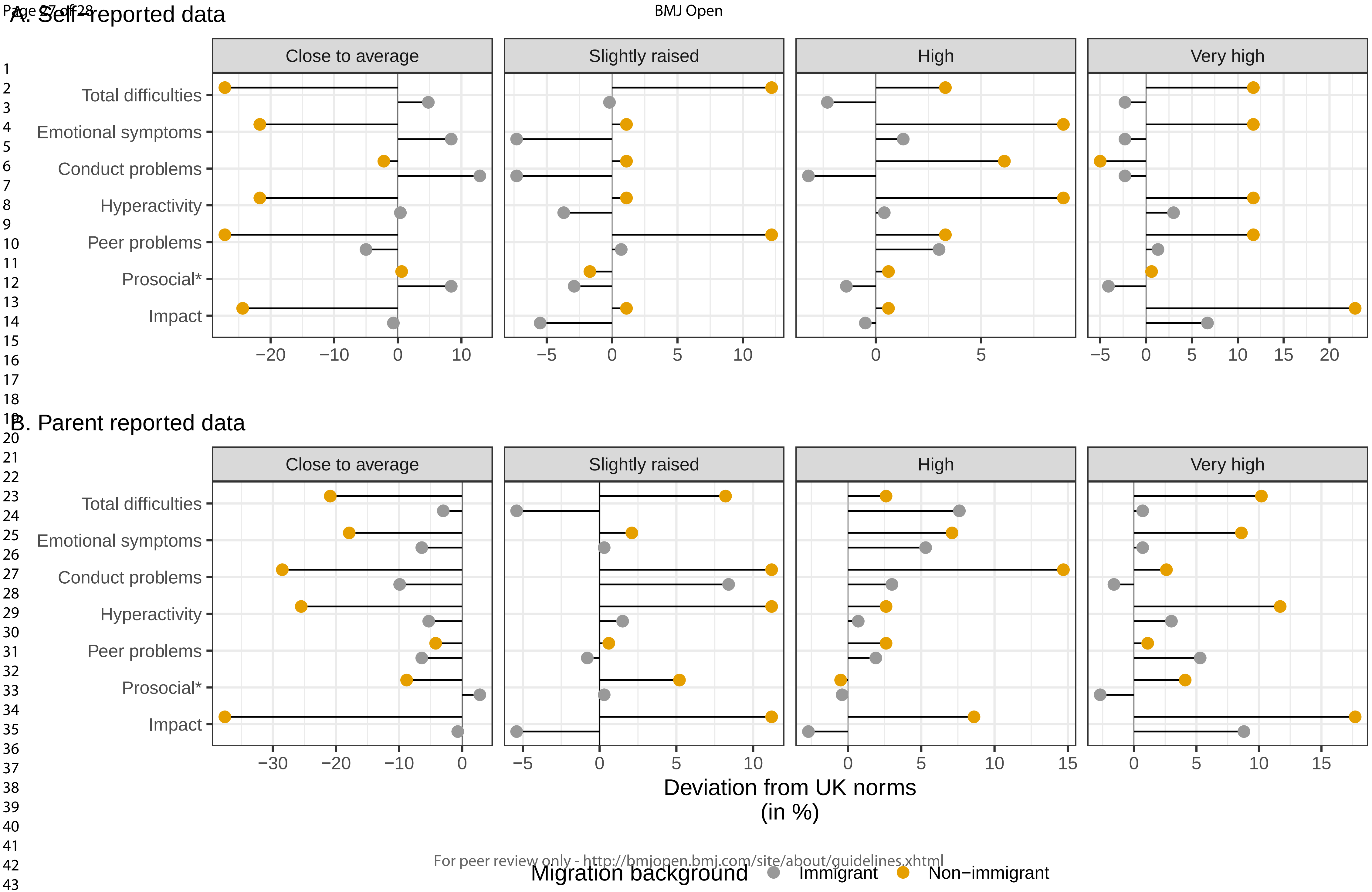
A. Self−reported data
Close to average
Slightly raised
High
Very high
Total difficulties
Emotional symptoms
Conduct problems
Hyperactivity
Peer problems
Prosocial*
Impact
−20 −10 0 10
−5 0 5 10
0 5
−5 0 5 10 15 20
B. Parent reported data
Close to average
Slightly raised
High
Very high
Total difficulties
Emotional symptoms
Conduct problems
Hyperactivity
Peer problems
Prosocial*
Impact
−30 −20 −10 0
−5 0 5 10
0 5 10 15
0 5 10 15
Deviation from UK norms
(in %)
Migration background
Immigrant
Non−immigrant

STROBE Statement—Checklist of items that should be included in reports of ***cross-sectional studies***

	Item No	Recommendation	Page No
**Title and abstract**	1	(*a*) Indicate the study's design with a commonly used term in the title or the abstract	2
		(*b*) Provide in the abstract an informative and balanced summary of what was done and what was found	2
**Introduction**			
Background/rationale	2	Explain the scientific background and rationale for the investigation being reported	3-4
Objectives	3	State specific objectives, including any prespecified hypotheses	4
**Methods**			
Study design	4	Present key elements of study design early in the paper	4
Setting	5	Describe the setting, locations, and relevant dates, including periods of recruitment, exposure, follow-up, and data collection	4
Participants	6	(*a*) Give the eligibility criteria, and the sources and methods of selection of participants	4
Variables	7	Clearly define all outcomes, exposures, predictors, potential confounders, and effect modifiers. Give diagnostic criteria, if applicable	5-6
Data sources/ measurement	8*	For each variable of interest, give sources of data and details of methods of assessment (measurement). Describe comparability of assessment methods if there is more than one group	5-6
Bias	9	Describe any efforts to address potential sources of bias	12
Study size	10	Explain how the study size was arrived at	4-5
Quantitative variables	11	Explain how quantitative variables were handled in the analyses. If applicable, describe which groupings were chosen and why	6
Statistical methods	12	(*a*) Describe all statistical methods, including those used to control for confounding	6
		(*b*) Describe any methods used to examine subgroups and interactions	6
		(*c*) Explain how missing data were addressed	6
		(*d*) If applicable, describe analytical methods taking account of sampling strategy	6
		(*e*) Describe any sensitivity analyses	6,11
**Results**			
Participants	13*	(a) Report numbers of individuals at each stage of study—eg numbers potentially eligible, examined for eligibility, confirmed eligible, included in the study, completing follow-up, and analysed	18
		(b) Give reasons for non-participation at each stage	na
		(c) Consider use of a flow diagram	na
Descriptive data	14*	(a) Give characteristics of study participants (eg demographic, clinical, social) and information on exposures and potential confounders	18
		(b) Indicate number of participants with missing data for each variable of interest	na
Outcome data	15*	Report numbers of outcome events or summary measures	20-22

For peer review only

Main results	16	(*a*) Give unadjusted estimates and, if applicable, confounder-adjusted estimates and their precision (eg, 95% confidence interval). Make clear which confounders were adjusted for and why they were included	22
		(*b*) Report category boundaries when continuous variables were categorized	na
		(*c*) If relevant, consider translating estimates of relative risk into absolute risk for a meaningful time period	na
Other analyses	17	Report other analyses done—eg analyses of subgroups and interactions, and sensitivity analyses	10
**Discussion**			
Key results	18	Summarise key results with reference to study objectives	9-10
Limitations	19	Discuss limitations of the study, taking into account sources of potential bias or imprecision. Discuss both direction and magnitude of any potential bias	11
Interpretation	20	Give a cautious overall interpretation of results considering objectives, limitations, multiplicity of analyses, results from similar studies, and other relevant evidence	9-13
Generalisability	21	Discuss the generalisability (external validity) of the study results	12
**Other information**			
Funding	22	Give the source of funding and the role of the funders for the present study and, if applicable, for the original study on which the present article is based	17

*Give information separately for exposed and unexposed groups.

Note: An Explanation and Elaboration article discusses each checklist item and gives methodological background and published examples of transparent reporting. The STROBE checklist is best used in conjunction with this article (freely available on the Web sites of PLoS Medicine at http://www.plosmedicine.org/, Annals of Internal Medicine at http://www.annals.org/, and Epidemiology at http://www.epidem.com/). Information on the STROBE Initiative is available at www.strobe-statement.org.

For peer review only
